# Sustainable Pyrotechnics: Combustion Behavior of B_4_C/Bi_2_O_3_ for Delay Compositions

**DOI:** 10.1021/acsomega.5c10964

**Published:** 2026-01-12

**Authors:** Danillo F. Vianna Cantini, Vojtěch Pelikán, Eva Schmidová, Jiří Pachman

**Affiliations:** † Institute of Energetic Materials, Faculty of Chemical Technology, University of Pardubice, Studentská 95, Pardubice 532 10, Czech Republic; ‡ Educational and Research Centre in Transport, Faculty of Transport Engineering, University of Pardubice, Studentská 95, Pardubice 532 10, Czech Republic

## Abstract

Understanding the combustion behavior of greener pyrotechnic delay compositions is key to developing more sustainable and efficient materials for practical applications. This study explores B_4_C, a high-reactivity fuel, and Bi_2_O_3_, an efficient oxidizer, as eco-friendly alternatives to traditional formulations. Thermodynamic calculations guided formulation design, predicting major combustion products. After formulation, the compositions were granulated, pressed at varying pressures (64–385 MPa), burned, and analyzed for their burning rates. Results show that increasing compaction leads to a progressive decrease in burning rates, as reduced porosity shifts combustion from convective to conductive. Particle size influences combustion up to a certain limit, while the granule size of the final composition alters the burning profile. The reaction unfolds in multiple stages, starting with a solid–solid preignition phase, then moving to a solid–liquid phase, and eventually reaching a gas stage. Complementary analyses of solid residues and combustion gases supported the proposed reaction pathway. These findings provide crucial insights into optimizing both performance and environmental impact reinforcing the viability of B_4_C/Bi_2_O_3_ formulations for practical applications.

## Graphical abstract

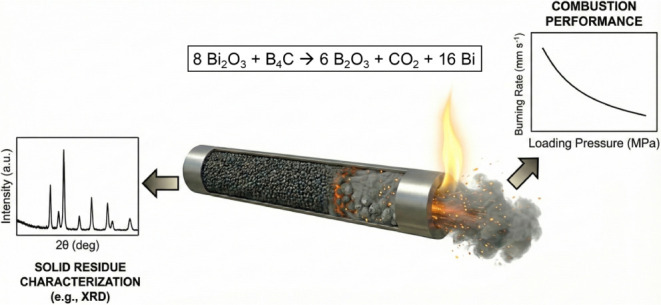

## 1 Introduction

Pyrotechnic delay compositions are formulations designed to create a specific delay time, determined primarily by their burning rate. These compositions are essential in timing energetic events and are used extensively in both civilian and military applications.^1^ This broad usage leads to diverse motivations for studying such materials. In civilian contexts, such as mining, the focus is typically on safety and cost-effectiveness.^2^ For military purposes, reliability and precision are key factors, often with stringent performance requirements.^3^

Pyrotechnic delay compositions are classified based on their combustion behavior into gassy or gasless types. In confined, sealed and obturated environments, gasless compositions are favored, to avoid gas buildup.^1,4^ However, in unconfined or vented systems, gassy compositions are not problematic. For example, in fireworks, where combustion gases freely dissipate,^1^ or in vented housings like hand grenades or signaling devices, gassy delays are acceptable.^5,6^

In gassy compositions, the burning rate is influenced by the loading (or consolidation) pressure. Hot gases generated by the chemical reaction penetrate gas channels (voids) in the material, igniting unreacted material through a process known as convective heating. As loading pressure increases, these voids are gradually reduced, which diminishes convection and subsequently lowers the burning rate. In contrast, for gasless compositions, burning is driven by conductive heating, where heat is transferred through direct particle-to-particle contact, leading to the opposite effect.^7–9^

Historically, up until World War II, black powder served as the foundation for most delay elements. Nowadays, it continues to be utilized for this function and others, but on smaller scales.^1^ Some more recent and traditional formulations for pyrotechnic compositions include fuels like silicon, boron, tungsten, zinc, iron, zirconium, and manganese. Examples of these are Si/Pb3O_4_,^8,10^ B/BaCrO_4_ ^8^, W/BaCrO_4_/KClO_4_,^5,6,11^ Zn/PbO_2_,^12^ Fe/BaO_2_,^13^ Zr/KClO_4_,^8^ and Mn/PbCrO_4_/BaCrO_4_.^14^

Currently, researchers are actively developing several new formulations for pyrotechnic delays. One of the main motivations for this is the need to transform these compositions into “greener” ones.^1,2^ This means that compositions that could cause problems for the environment or human health should be avoided. To achieve this sustainability, the identification and replacement of known or potentially harmful, toxic, and carcinogenic compounds is vital.^10^ Heavy metals, persistent organic pollutants, toxic gases, volatile organic compounds and known causers of human health problems are examples of such substances to be removed.^2,5,15^

Specifically, there are several attempts to replace lead, barium, chromates, and perchlorates.^15^ The reason is that most of the established delay formulations used today contain these substances.^2,5^ Lead, barium and chromium (contained in chromates) are heavy metals and are known to cause serious health problems in human, specially to nervous, cardiovascular and respiratory systems.^5,16^ Perchlorates are well-documented substances and are used in various pyrotechnic applications. However, they are widely recognized as contributors to thyroid issues.^5^

From the above, it is clear that modern strategies in pyrotechnics are increasingly focused on developing new materials that prioritize both efficiency and sustainability. This shift imposes numerous restrictions, meaning that formulations must be designed with precise planning and thorough investigation to meet both performance and environmental standards.

Boron could be considered the ideal fuel for pyrotechnic reactions due to its high energy content. However, it presents combustion issues for its limited reactivity due to boron oxide layer and boron agglomerates formation upon heating.^17^ The boron oxide layer acts as a barrier, hindering oxygen (O) diffusion and consequently limiting the oxidation of boron.^18^

Boron carbide (B_4_C) is a natural candidate for combustion processes due to its lower ignition point, which is attributed to its higher reactivity. This reactivity stems from the diffusion of carbon (C) particles, which oxidize into gaseous products. These gaseous products create diffusion channels that penetrate the boron oxide layer, breaking down the layer and agglomerates, thereby further improving the reaction.^18,19^ B_4_C presents better combustion properties, with better burning stability and reliable self-propagation, resulting in better combustion efficiency.^17^

Bismuth trioxide (Bi_2_O_3_) is an attractive oxidizer for pyrotechnic delay compositions due to several advantageous properties. Its high oxygen content and efficient oxygen release facilitate sustained combustion, while its favorable thermal properties, including a relatively low melting and decomposition temperature, ensure that oxygen is available at precisely the right moment to support a controlled reaction.^20^ Additionally, Bi_2_O_3_ high density allows for compact formulations, promoting consistent and reliable burn rates. Also, it has a low heat of formation (approximately −582 kJ/mol), which is crucial to minimize the energy required to initiate its decomposition. This enhances the overall reaction efficiency, allowing Bi_2_O_3_ to rapidly release oxygen without consuming excessive energy, thus ensuring sustainable and stable combustion.^8^

In addition to their technical advantages, Bi_2_O_3_ and B_4_C offer a significant environmental benefit: they are considered more eco-friendly compared to other materials commonly used in pyrotechnic compositions. Bi_2_O_3_ serves as a less toxic alternative to traditional oxidizers like lead-based compounds, reducing the risk of environmental contamination and exposure to harmful heavy metals.^1,8,16^ Similarly, B_4_C contributes to cleaner combustion, producing fewer pollutants and minimizing the environmental impact.^5^ This makes both materials not only effective but also greener choices for sustainable pyrotechnic applications.

Horiuchi^18^ investigated the B_4_C/Bi_2_O_3_ reactivity using loose powder in acrylic tubes, providing useful initial insights but without addressing application-specific conditions. In contrast, this study explores the burning behavior of compacted B_4_C/Bi_2_O_3_ mixtures, prepared with binder, granulated, and pressed near theoretical maximum density (TMD), reproducing their intended use in pyrotechnic delays.

This approach enabled the development of burning rate curves as a function of loading pressure, offering a deeper and more practical understanding of how density, particle size, and granulation affect ignition and combustion. Complementary analyses of gases and residues help elucidate the underlying combustion mechanism.

By bridging the gap between preliminary reactivity studies and practical implementation, this work prioritizes the exploration of low toxicity materials and cleaner manufacturing, improving processability and evaluating the burning behavior of the compositions. This allows for more precise insights into the mixture’s behavior in engineered systems, like delays, while promoting more sustainable practices through efficient material use.

## 2 Experimental Section

### 2.1 Materials

Boron carbide (<10 μm, 99+%, Alfa Aesar) and bismuth­(III) oxide (99+%, VWR Chemicals) were used as fuel and oxidizer, respectively. Particle size distributions were measured using a Malvern Mastersizer 3000 (Malvern Panalytical), yielding Dv(50) of 5.93 μm for B_4_C and 9.45 μm for Bi_2_O_3_. BET surface areas, measured using a Nova Station A (Quantachrome Instruments), were 3.287 m^2^/g and 0.401 m^2^/g, respectively. Bi_2_O_3_ and B_4_C were dried at 60 °C and sieved into <32 μm, 32–63 μm, and >63 μm fractions. B_4_C showed no retention above 32 μm, while Bi_2_O_3_ appeared in all fractions. Although Malvern analysis detected only finer particles for Bi_2_O_3_, the presence of larger ones suggests agglomeration, a phenomenon commonly reported for this material.^21–23^ The ultrasound function of the Malvern system effectively dispersed these clusters, revealing the true particle size distribution. Poly­(vinyl alcohol) (PVA, Fichema) 16%, in water, and gum arabic (in-house) were tested as 1 wt % binders. Ethanol (99.9%, Merck) and water were used as green solvents. Visco fuses containing black powder (3 mm diameter, 3 cm/s burning rate) and available in-house were used for ignition of the delays.

### 2.2 Experimental Planning

The initial batches followed the balanced thermite reaction between B_4_C and Bi_2_O_3_, corresponding to 4.25 wt % B_4_C and 95.75 wt % Bi_2_O_3_, assuming complete oxidation/reduction of reactants, as represented in eq 1 Thermodynamic predictions were performed using the REAL WIN software to support/validate the reaction.^24^

1 $$8{\mathrm{Bi}}_{2}O_{3}+3B_{4}C↔6B_{2}O_{3}+3CO_{2}+16\mathrm{Bi}$$

Granulation was conducted using ethanol or water to minimize toxicity. Based on their compatibility with these solvents, two sustainable and purely organic binders, PVA and gum arabic were tested as green binders, at 1 wt % of the total mixture. The initial batches (Table 1) burned successfully in open air when unpressed, consistent with Horiuchi’s findings,^18^ but failed to ignite when lightly pressed into delay elements, even at 19 MPa.

**Table 1:** Preliminary Experiments (with 4.25% B_4_C)

Batch	Bi_2_O_3_ size	Binder	Solvent	Granulation sieve
A0	32 < *x* < 63 μm	PVA	Ethanol	600 μm
A1	32 < *x* < 63 μm	Gum Arabic	Ethanol	600 μm
A2	32 < *x* < 63 μm	PVA	Water	600 μm

These results indicated that stoichiometric formulations were unsuitable for compacted conditions. To enhance ignitability, the fuel content was increased to 8.5 wt % B_4_C (91.5 wt % Bi_2_O_3_), and PVA was selected as the sole binder based on better processability. This new formulation was adopted for all subsequent batches.

To establish a scientific baseline for combustion characteristics, both formulations were tested in a combustion chamber to analyze pressure evolution, gas-phase and solid products.

Other processing parameters were also refined based on handling and performance observations:

1. The particle size of Bi_2_O_3_ was systematically varied to assess its impact on burning behavior.
2. Water replaced ethanol after batch B2 due to better dough consistency.
3. A 200 μm postgranulation sieve was added from batch B3 onward.
4. Batch B6 used a 1 mm sieve to evaluate the effect of larger granules.

These modifications are summarized in Table 2, which shows the revised manufacturing conditions for the new batches.

**Table 2:** New Batches Manufacturing Conditions (with 8.5% B_4_C)

Batch	Bi_2_O_3_ size	Solvent	Postgranulation sieve	Granulation sieve
B1	32 < *x* < 63 μm	Ethanol	-	600 μm
B2	32 < *x* < 63 μm	Ethanol	-	600 μm
B3	*x* > 63 μm	Water	63 μm,200 μm	600 μm
B4	32 < *x* < 63 μm	Water	200 μm	600 μm
B5	*x* < 32 μm	Water	200 μm	600 μm
B6	32 < *x* < 63 μm	Water	200 μm	1 mm

### 2.3 Delay Composition Preparation

Compositions were prepared by weighing B_4_C and Bi_2_O_3_ using a four-digit precision electronic scale. The materials were then mixed using the sieve brush technique, passing through a 500 μm sieve at least four times to ensure homogeneity. The binder (PVA or gum arabic) was dissolved in the solvent (ethanol or water), and the mixture was stirred until a homogeneous suspension was achieved. The dry components were added to this solution and mixed into a uniform dough.

### 2.4 Granulation Varied by Solvent

- Ethanol-based batches: The dough was gradually dried and brushed through a 600 μm sieve while still pliable—dry enough not to form wet granules but not too brittle to become dusty powder.
- Water-based batches: The dough was oven-dried at 40 °C, checked every 5 min, and sieved once it reached the proper consistency.

### 2.5 Drying

After granulation, all batches were dried at 60 °C until mass stabilized. Samples were then stored in sealed containers to prevent moisture reabsorption. Thermogravimetric Analysis (TGA) and Differential Scanning Calorimetry (DSC) confirmed sample stability, in agreement with prior findings for Bi_2_O_3_/B_4_C^18^ and PVA.^25^

### 2.6 Pressing

Each composition was pressed in four 180 mg increments into 25.3 mm steel delay tubes (⌀ 3.15/5.15 mm), with 20 s consolidation time for the each increment. A distance sensor on the punch measured the final column height. Applied forces ranged from 500 to 3000 N (64–385 MPa), achieving densities between 61% and 88% of the theoretical maximum density (TMD), increasing progressively with pressure. Samples were burned immediately after pressing.

### 2.7 Burning

Delay elements were ignited via visco fuses. Burn tests were recorded using a 240-fps slow-motion camera. The delay time was measured by selecting the initial frame in which the flame from the delay composition became visible at the back end, which provided a clear contrast with the burning behavior of the visco fuse. The delay time was determined by identifying the first frame showing the flame at the opposite end of the delay element. Burning rate was calculated as the delay length divided by total burn time.

Tests were conducted in open air under ambient conditions (18–28 °C, 40–70% RH) to eliminate external pressure as a variable. While no standardized method exists for pyrotechnic delay burning rate testing, this approach ensured repeatability and reliable comparison between batches.

### 2.8 Material Characterization

Particle and granule size distribution were measured with a Malvern Mastersizer 3000 (Malvern Panalytical). Scanning Electron Microscopy (SEM) was carried out with a TESCAN MIRA (TESCAN) to examine morphology and confirm particle size. A 40 cm^3^ combustion chamber was used to analyze pressure profile, quantify solid residues, and collect combustion gases for further analysis. Fourier Transform Infrared Spectroscopy (FTIR) was performed using a Nicolet iS50 FT-IR (Thermo Scientific) and standardized nitrogen (N_2_) background to identify gas-phase products. Thermal stability and water content assessments were conducted using TGA (NETZSCH TG 209 F3 Tarsus) and DSC (NETZSCH DSC 200 F3 Maia). Energy Dispersive X-ray Spectroscopy (EDS) was performed using a Bruker Quantax 200 (Bruker) for semiquantitative chemical composition analysis of the combustion residues. X-ray diffraction (XRD) analyses were performed using a Rigaku MiniFlex diffractometer for complementary phase identification, while surface chemical states were characterized by X-ray photoelectron spectroscopy (XPS) using a Scienta Omicron ESCA2SR system.

## 3 Results and Discussion

### 3.1 Malvern and SEM

Malvern analyses confirmed the particle size distributions of the granulated materials, which are crucial for burn rate uniformity. Unsieved compositions (B1–B3) showed broad, poorly defined distributions, while those sieved through a 200 μm mesh (B3–B6) exhibited bimodal profiles with a main peak near the sieve cutoff and a secondary peak between 7–20 μm. For B3–B5, Dv(50) values ranged from 193 to 243 μm—lower than the 600 μm sieve size—likely due to partial fragmentation during handling or limitations of the laser diffraction model, which assumes spherical particles and may underestimate irregular shapes. The fine mode suggests residual fines from raw materials or postprocessing. B6, granulated with a 1 mm sieve, also displayed a bimodal distribution, with a Dv(50) of 390 μm, consistent with its coarser granulation.

SEM analyses were performed to characterize the morphology and validate experimental observations. Secondary Electrons (SE) and Backscattered Electrons (BSE) imaging were employed. Figure 1 shows raw Bi_2_O_3_ and B_4_C, where BSE contrast clearly distinguishes heavier Bi_2_O_3_ (brighter) from lighter B_4_C (darker), confirming their irregular morphology and fine particle size—factors that influence granule packing and combustion.

**Figure 1:**
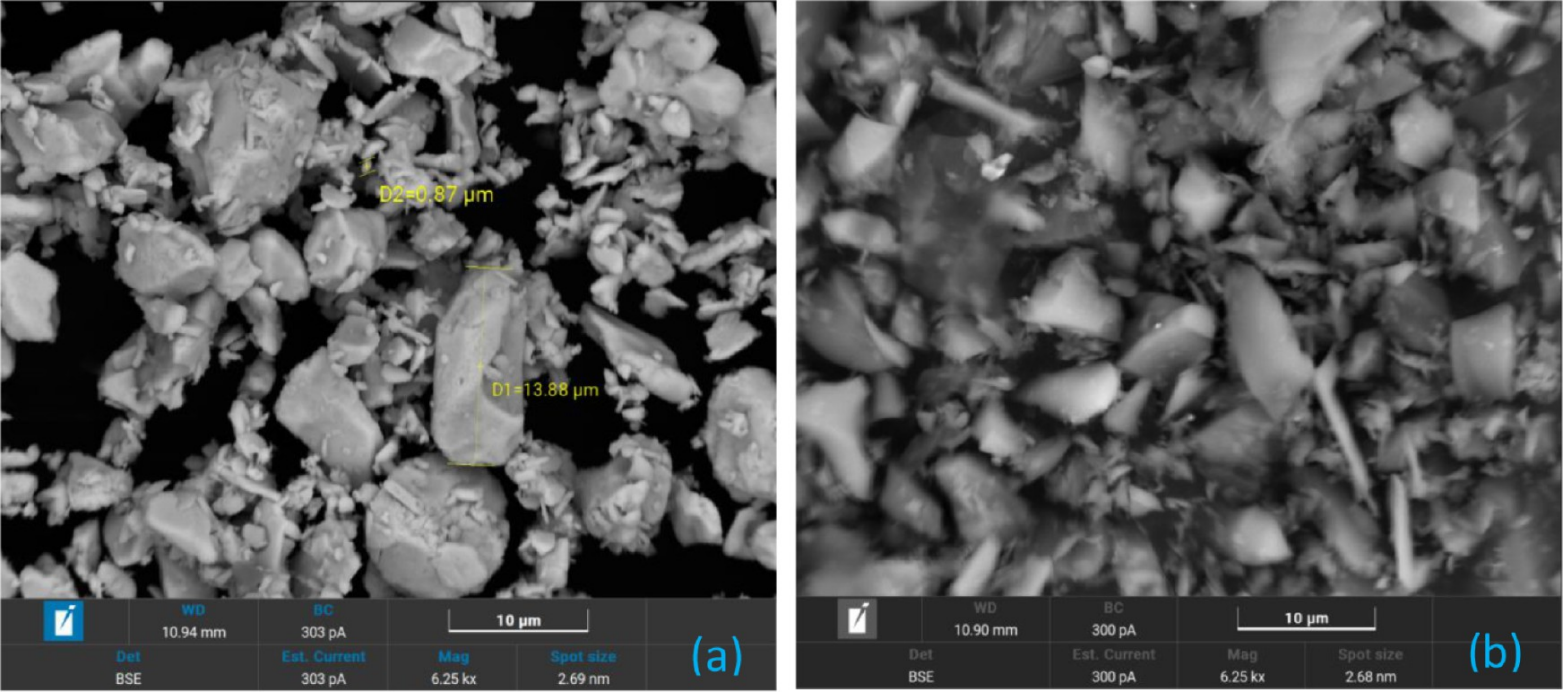
Raw Bi_2_O_3_ (a) and B_4_C (b) SEM analysis with BSE technique.

Figure 2 (BSE) and Figure 3 (SE) show SEM images of B4 granules before pressing. Due to B4’s similarity with other batches, these observations are broadly representative. The images reveal strong cohesion between particles, indicating effective binder action and granulation, which is expected to improve pressing efficiency, minimizing inconsistencies in burning rates.

**Figure 2:**
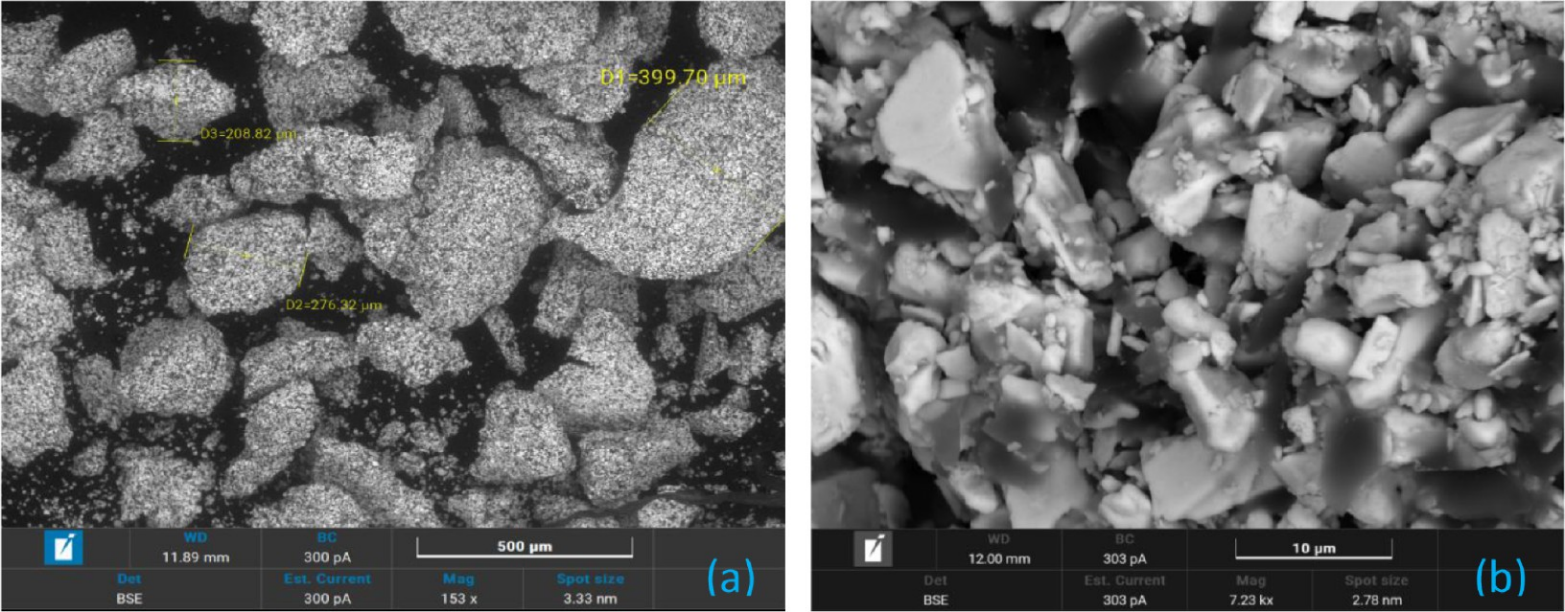
SEM analysis of B4 granules before pressing using the BSE mode: (a) low-magnification (153 ×) overview of the granulated structure; (b) high-magnification (7.23 k×) view of individual granules.

**Figure 3:**
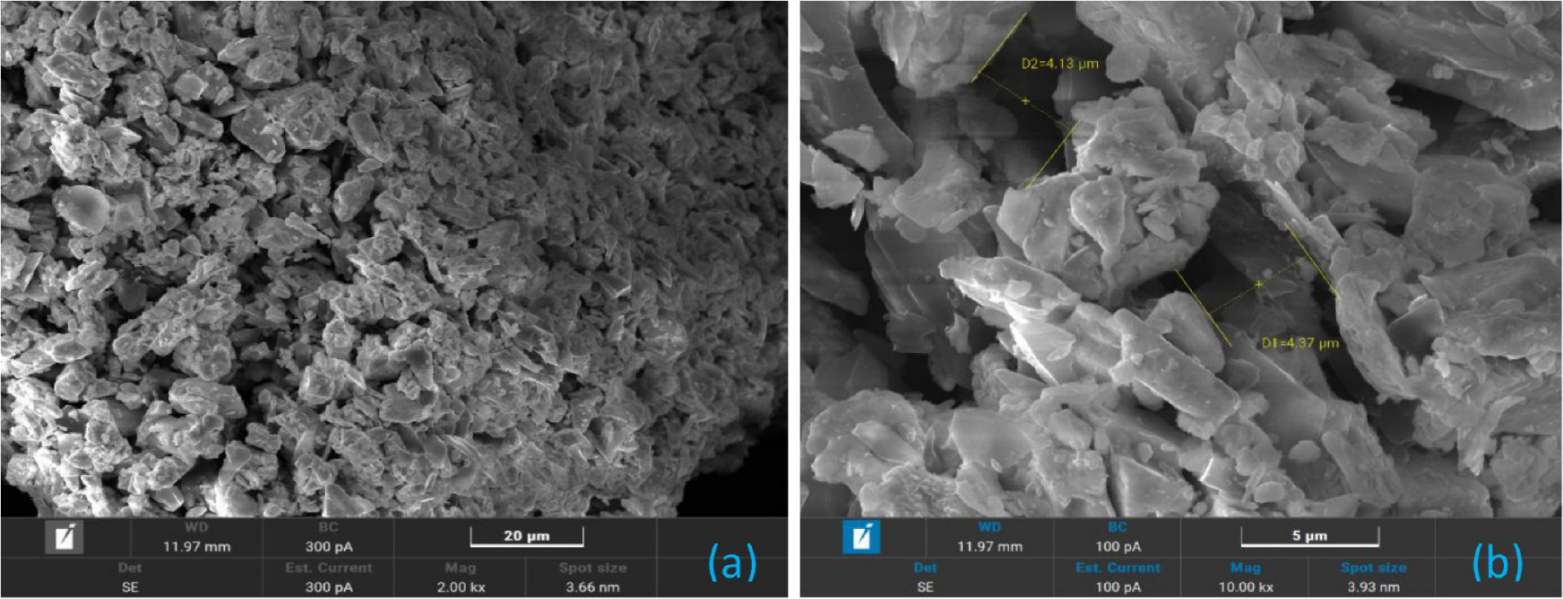
SEM analysis of B4 granules before pressing using the SE mode: (a) low-magnification (2 k×) overview of the granulated structure; (b) high-magnification (10 k×) view of individual granules.

Figure 2a shows granules >200 μm, consistent with sieving and Malvern results. Figure 2b confirms expected Bi_2_O_3_/B_4_C separation, consistent with mechanical mixing. Figure 3 reveals tightly packed structures with minimal interstitial spaces (<5 μm), important for understanding heat transfer and burning behavior. Loose particles likely result from postprocessing handling or SEM sample preparation.

Building on, the next step was to investigate how processing parameters influence the burning rate in operational conditions. The following experiments were designed to assess the impact of formulation adjustments, particle size variations, and granulation on combustion performance for delay applications.

### 3.2 Burning Rate Results of Preliminary Experiments

All batches (A0–A2), stoichiometric compositions, burned reliably when pressed in open environment, in the bulk form of loose powders. However, when pressed with very low loading pressures of 19 MPa, the system was unable to ignite. This result points out the first difference from Horiuchi’s^18^ work and highlights the importance of testing conditions for end-user applications.

Given these initial ignition failures in the stoichiometric compositions, the formulation was adjusted to a fuel-rich ratio to enhance ignition reliability and burning performance.

### 3.3 Burning Rate Results of Ethanol-Based Batches

Following the fuel ratio adjustments, the compositions no longer exhibited ignition issues, demonstrating the effectiveness of these modifications. With ignition consistency established, the next step was to evaluate the burning rate under different conditions.

B1 utilized Bi_2_O_3_ particles with sizes between 32 and 63 μm and ethanol as the solvent. The compositions were easily ignited by the visco fuse, demonstrating reliable initiation and sustaining steady combustion throughout the process without quenching. In five repetitions, it demonstrated good reproducibility at a loading pressure of 64 MPa, with burning rates ranging from 7.45 to 7.72 mm s^–1^, averaging 7.54 mm s^–1^, and a standard deviation of 0.12 mm s^–1^. This batch produced stable results, indicating consistent performance under these conditions.

B2 was produced under the same conditions as B1. When subjected to a higher loading pressure of 128 MPa, this batch also demonstrated good reproducibility, with burning rates ranging from 7.77 to 7.98 mm s^–1^, averaging of 7.83 mm s^–1^, and a standard deviation of 0.10 mm s^–1^, based on five repetitions at this new pressure. These results initially suggested that the ethanol-based process could deliver consistent performance when the pressing conditions were kept constant. However, when this same batch was later used to evaluate the effect of different loading pressures (64–385 MPa), the data showed a scattered trend, peaking at around 256.6 MPa, as represented in Figure 4.

**Figure 4:**
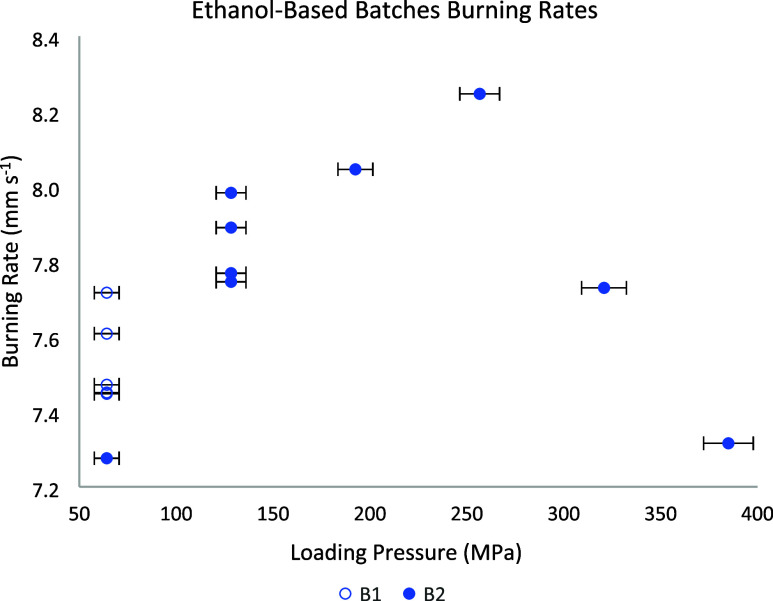
Ethanol-based batches burning rate vs loading pressure.

Although the data from B1 and B2 were individually consistent, the overall trend exhibited a surprising scatter, deviating from the expected behavior. Tested fits yielded poor goodness-of-fit and structured residuals, confirming that the data do not follow a monotonic trend and that imposing a parametric law would be misleading. According to Horiuchi,^18^ such formulations are anticipated to produce a significant amount of gas, indicating that convective burning should be prominent under these experimental conditions. As the loading pressure increases, the reduction in air spaces suppresses convective heat transfer, favoring a transition to predominantly conductive burning.^8,9^ Consequently, higher loading pressures are expected to result in progressively lower burning rates, up to a limit, following an asymptotic profile, as demonstrated by examples of Pyrodex (gassy composition) in Kosanke’s^7^ work.

Moreover, the manufacturing process using ethanol proved impractical due to poor binder distribution. Ethanol was initially selected because its faster evaporation was expected to facilitate granulation and shorten the drying time. Upon adding ethanol to the pyrotechnic mixture, the areas where the solvent made contact clumped together almost immediately, while the rest of the mixture did not integrate well. This resulted in a heterogeneous composition, with some sections of the dough appearing rubbery and others brittle. The lack of uniformity raises concerns about the effectiveness of ethanol as a solvent in this process, suggesting that the solvent might not be dispersing evenly across the mixture. This could lead to uneven distribution of components and, ultimately, inconsistent performance during combustion.

For this reason, water, which is reported to be the best solvent for PVA, was selected for use in the following batches. Preliminary dispersion tests confirmed that distilled water allowed a gradual and uniform distribution of the binder solution, without premature clumping or separation.

### 3.4 Burning Rate Results of Water-Based Batches

Besides replacing ethanol with water as the solvent, it was intended to investigate how variations in oxidizer particle size influence burning behavior. To systematically evaluate these factors, three compositions were analyzed: B3 (*x* > 63 μm), B4 (32 μm < *x* < 63 μm), and B5 (*x* < 32 μm). This approach isolates the effect of particle size on burning rate while maintaining consistent processing conditions to ensure comparable results.

B3 was prepared accordingly, with adjustments in the drying and granulation process to accommodate the solvent change. Since PVA is fully water-soluble, the mixture becomes significantly more homogeneous. Although water takes longer to evaporate, which might initially seem like a disadvantage, the extended drying time offers a benefit: it provides sufficient time to work the dough through the sieve without it drying out and becoming brittle.

One additional concern with using water was the potential for residual moisture. To address this, TGA and DSC analyses were performed for the B3 composition, as shown in Figures 5 and 6. The TGA graphs show a humid sample immediately after the granulation process and a dry sample following 4 h of drying at 60 °C. The humid sample exhibits a clear mass loss associated with water evaporation, while the dry sample shows no detectable mass loss, confirming the absence of residual moisture and the stability of the sample after drying. The DSC analysis of another sample of the dried composition further supports this conclusion, by showing no thermal events (e.g., phase changes or decomposition) in the temperature range analyzed. These results confirm the effectiveness of the drying process. Based on these findings, all subsequent batches were dried in an oven at 60 °C for 4 h. After this period, no mass loss was observed in any sample, ensuring consistency across preparations.

**Figure 5:**
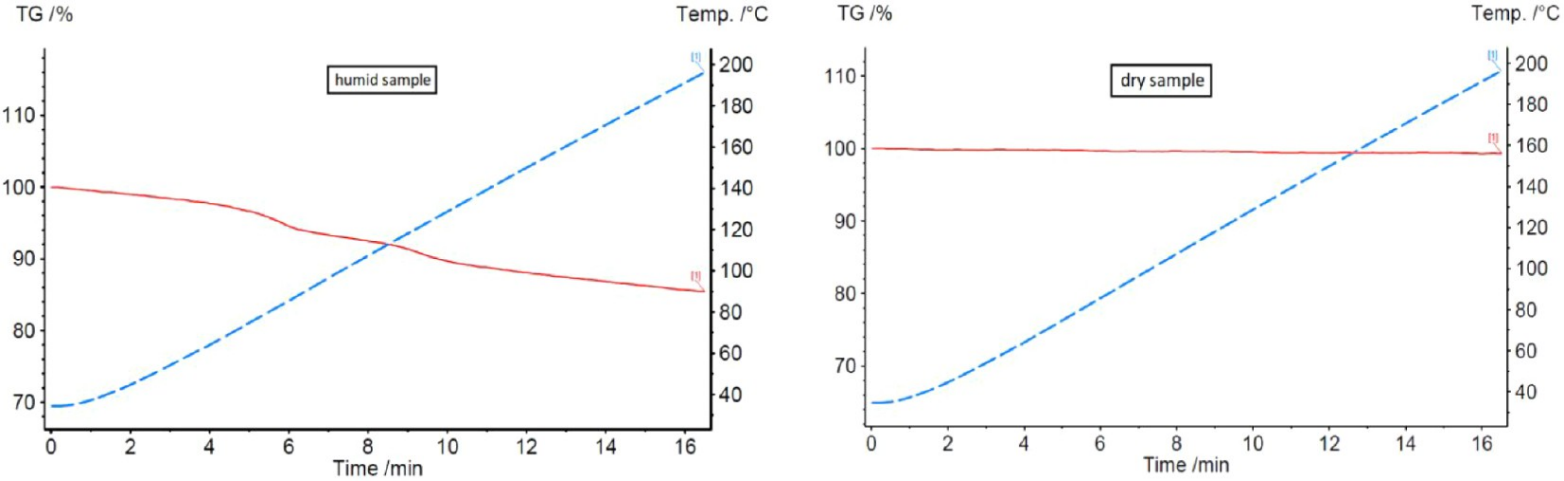
TGA curves of B3 humid and dry samples.

**Figure 6:**
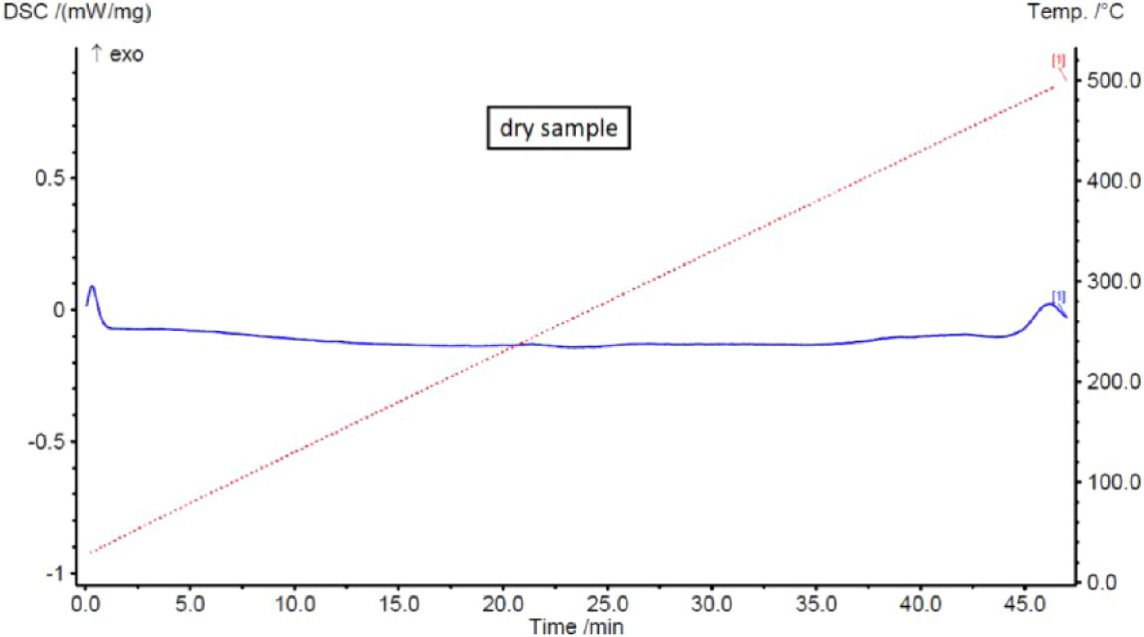
DSC curve of the B3 dry sample.

Furthermore, to reduce the dust in the final product, the postgranulation sieving effect on burning rate was evaluated using three compositions: unsieved, sieved with particles larger than 63 μm, and sieved with particles larger than 200 μm. The results for each one of the compositions are shown in Figure 7, with error bars for loading pressure indicating the measurement uncertainty during the pressing process. Although the burning rate ranges were similar across the different samples (9.90 to 8.49 mm s^–1^), the lower scatter of data from 63 μm onward highlights the enhanced consistency and performance with finer sieving.

**Figure 7:**
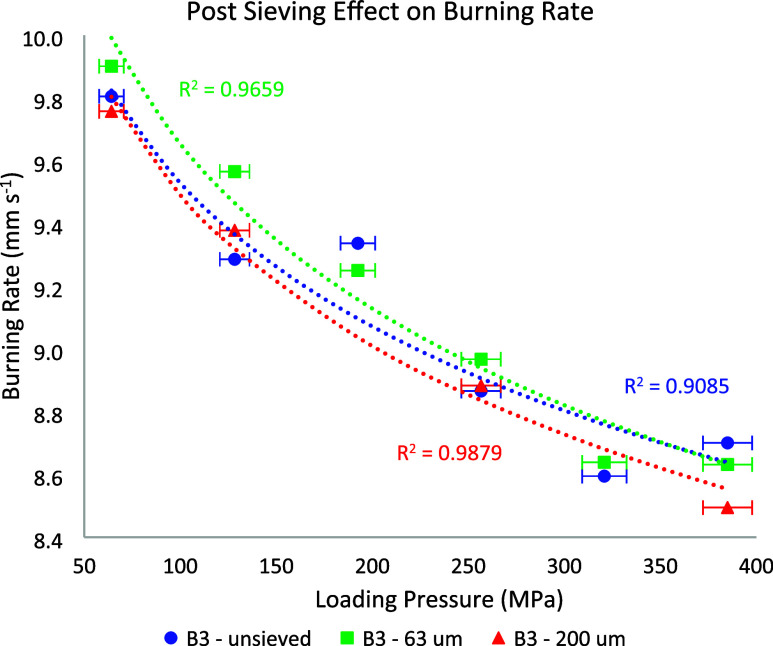
Burning rate vs loading pressure of the three B3 compositions, highlighting the effect of postsieving.

The observed burning rate results align more closely with the expected behavior of this type of composition and support the predicted occurrence of gas production. The experimental data were fitted using a logarithmic decay function, which adequately describes the asymptotic decrease in burning rate with increasing compaction pressure and was selected because it reflects the physical trend associated with progressive pore closure and reduced gas flow within the compacted mixture.

Overall, the use of water not only improved the binder compatibility but also enhanced mixture homogeneity, leading to more consistent combustion behavior and easier granulation. Sieving effectively reduced dust, improving the pressing process. The removal of fine particles prevented microgaps between the pressing punch and the wall of the delay element from being obstructed by dust raised by the small displacement of trapped air during punch descent. This allowed for smoother operation and more precise interaction between components. Therefore, modifications were concluded to greatly enhance the results, and all subsequent samples were sieved accordingly to ensure improved consistency and performance.

B4 was prepared similarly to B3 with exception of the Bi_2_O_3_ particle size, which was smaller (32 μm < *x* < 63 μm). The composition was also completely sieved in a 200 μm sieve. The burning rates (shown in Figure 8) consistently varied between 9.27 and 8.23 mm s^–1^, slightly lower than those observed in B3. The burning rate profile followed the expected trend of decreasing rate with increasing load. Although smaller particles are generally expected to yield higher burning rates in bulk form, due to their greater surface area, this trend may not hold under pressed conditions. Larger Bi_2_O_3_ particles, such as those used in B3, can generate a more open structure, with more voids and greater porosity within the mixture due to lower packing efficiency.^26,27^ As mentioned, these voids enhance convective heat transfer and accelerate the overall reaction rate.^8,9^ Additionally, smaller particles are more prone to agglomeration during processing and burning, which may reduce their effective surface area and particle-to-particle contact.^28–30^

**Figure 8:**
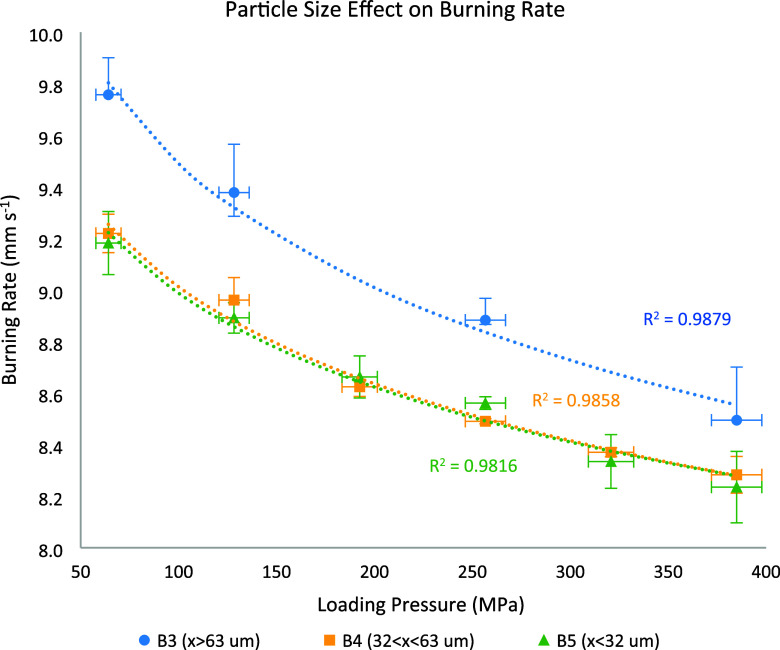
Burning rate vs loading pressure comparison of B3, B4, and B5, highlighting the particle size effect.

B5 was utilized to evaluate the burning rate of particles smaller than 32 μm. The burning rates observed ranged from 8.14 to 9.27 mm s^–1^, following the characteristic profile. These results closely match those of B4, indicating that at these size levels (*x* < 63 μm) similar packing efficiency is achieved, and particle size alone does not appear to significantly influence the burning rate. A comparison between these three batches is shown in Figure 8. The error bars for loading pressure indicate the measurement uncertainty during the pressing process. For the burning rate, the error bars for B3 represent the range of all experiments within this batch. For B4 and B5, the error bars indicate the standard deviation from experiments.

The dependence of oxidizer particle size on combustion behavior suggests that the reaction begins in a solid–solid regime. In this stage, particle size critically affects packing, porosity, and contact between reactants, directly influencing reactivity and combustion efficiency. If the reaction started primarily in a liquid-phase—with early melting of Bi_2_O_3_—these effects would be less pronounced, as the molten oxidizer would coat B_4_C particles uniformly, facilitating oxygen and heat transfer while potentially obstructing gas flow, reducing the impact of porosity and particle size variations.^31^

Thermal analysis data from Horiuchi^18^ further support this hypothesis: exothermic peaks were observed at 536 °C and 577 °C, well below the Bi_2_O_3_ crystallinity transition (∼730 °C),^20^ its melting point (∼817 °C), and the onset of mass loss in TGA, indicating reaction initiation in the solid state.

This aligns with Hardt’s^32^ description of the preignition step in solid–solid reactions, where localized contact between particles triggers exothermic reactions that generate heat and gas, which propagate through porous channels—especially in mixtures with larger particles—enhancing heat and mass transfer.

These findings support a progressive mechanism: the reaction starts as solid–solid, then shifts to solid–liquid as Bi_2_O_3_ (mp 817 °C), B_2_O_3_ (mp 450 °C), and Bi (mp 271 °C) melt, and finally involves gas-phase transport as volatile products form. Gaseous species like CO_2_, CO (from carbon oxidation), and bismuth vapor (as temperatures approach Bi’s boiling point, ∼1564 °C) enhance convective heat transfer. The presence of bismuth vapor is consistent with the reaction’s exothermicity and likely localized temperature peaks. Altogether, this multistep mechanism builds upon Horiuchi’s^18^ observation of condensed-phase initiation and reinforces the role of particle size in influencing burn rate and efficiency, particularly under varied compaction conditions.

B6 was included in this study to evaluate the influence of granulation sieve size. While B4 and B6 share the same particle size distribution, B4 was granulated using a 600 μm sieve, whereas B6 used a 1 mm sieve. The burning rate results are shown in Figure 9. The error bars for loading pressure represent the measurement uncertainty during pressing, while for the burning rate, they indicate the standard deviation of each batch.

**Figure 9:**
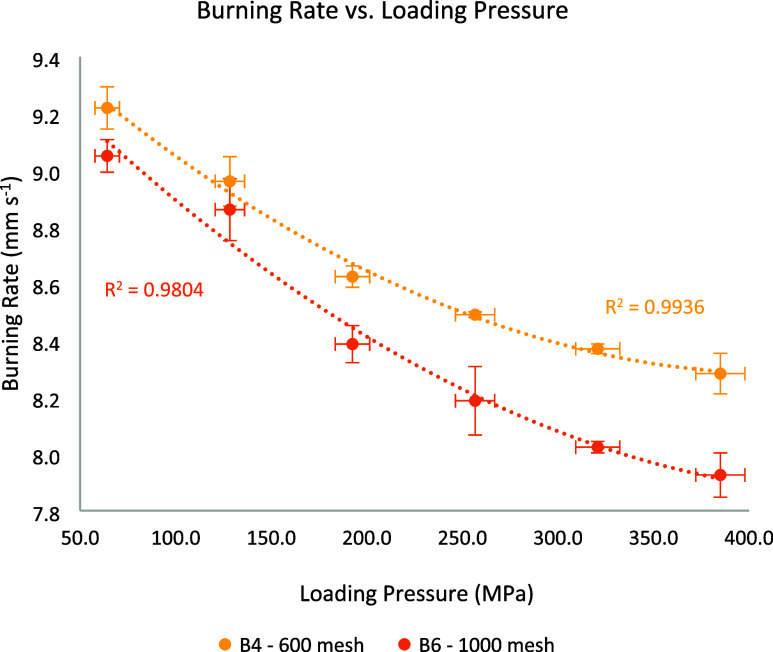
Burning rate vs loading pressure comparison of B4 and B6, highlighting the granulation effect.

For B6, at lower loading pressures, its burning rate was closer to B4, but as the pressure increased, the reduction in burning rate was steeper. This behavior can be explained by the larger granules produced from the 1 mm sieve. Under lower pressures, these granules likely increase porosity, promoting enhanced convective burning by allowing more gases and heat flow between particles, which increased the burn rate. As the loading pressures increased, the voids were compressed, reducing gas flow pathways and causing the burning rate to decrease.

Overall, the burning rates in this study ranged from 9.90 to 8.14 mm s^–1^, significantly lower than those reported by Horiuchi^18^ (∼20 mm s^–1^), likely due to differences in testing conditions—loose powder versus compacted samples. While Horiuchi observed combustion even in stoichiometric and fuel-lean compositions, here, only nonpressed stoichiometric samples burned, failing after compaction. These differences underscore the practical relevance of evaluating B_4_C/Bi_2_O_3_ mixtures under application-specific conditions, where granulation and compaction strongly affect combustion. Following these applied observations, a more fundamental analysis was carried out using combustion chamber tests and thermodynamic simulations to better understand the reaction pathway.

### 3.5 Combustion Chamber Experiment

A combustion chamber test was performed to obtain pressure-time evolution data, quantify solid residues, and collect combustion gases for analysis. For this experiment, both materials were burned in bulk form inside the combustion chamber. The maximum pressures recorded were 12 bar for a stoichiometric composition (A2) and 18 bar for a fuel-rich one (B4). The percentage of solid residues collected after the experiments was 90% for the stoichiometric composition and 85% for the fuel-rich one. These first results corroborate the expected gassy behavior of these compositions and further explain the burning rate dependence on loading pressure.

These experimental findings also provided essential boundary conditions for thermodynamic simulations, ensuring a realistic representation of combustion behavior.

### 3.6 Thermodynamic Calculations

Accurately predicting the exact quantities of combustion products is challenging due to the inherent complexity of modeling pyrotechnic reactions. To address this, thermodynamic simulations were conducted under systematically chosen boundary conditions, designed to account for both underestimation and overestimation of confinement and heat losses. The presence of air in the chamber’s free volume was considered in the calculations to improve accuracy. The oxygen balance was determined by the software to be −11.21% for the stoichiometric composition and −20.83% for the fuel-rich one. Table 3 summarizes the range of predicted combustion products, including their mass and molar fraction, condensed phase percentage, and adiabatic temperature.

**Table 3:** Predicted Products of Combustion of Both Types of Compositions, Condensed Amounts, and Adiabatic Temperatures

	Stoichiometric Composition		Fuel-Rich Composition	
Expected Products^a^	Mass Fraction	Molar Fraction	Mass Fraction	Molar Fraction
Bi (c)	0.536–0.667	0.368–0.444	0.586–0.668	0.347–0.390
B_2_O_3_ (c)	0.090–0.093	0.179–0.192	0.100–0.102	0.176–0.182
B_4_C (c)	0	0	0.030–0.031	0.067–0.069
BN (c)	0	0	0.014	0.069–0.070
Bi (g)	0.172–0.302	0.078–0.14	0.133–0.216	0.053–0.086
CO (g)	0.024–0.025	0.122–0.124	0.029–0.039	0.170–0.173
CO_2_ (g)	0.014	0.045–0.048	0	0
N_2_ (g)	0.008	0.040–0.041	0	0
H_2_ (g)	∼0	0.012–0.014	∼0	0.049–0.051
H_2_O (g)	0.003	0.025–0.027	0	0
B_2_O_2_ (g)	0	0	0.002–0.006	0.013–0.015
B_2_O_3_ (g)	0.003	0.003–0.006	0.001–0.002	0.002–0.003
HBO (g)	0	0	0.001	0.005–0.006
HBO_2_ (g)	0.013–0.015	0.043–0.049	∼0	0.001–0.002
Mass of Condensed Products (%)	62.9–75.7		78.17–81.35	
Adiabatic Temperature (°C)	1,808–2,080		1,850–2,007	

a (c)—Condensed; (g)—gas.

By integrating experimental data from the combustion chamber tests, the simulations provided a more representative depiction of the system’s behavior, enabling a qualitative comparison of the major combustion products. A detailed quantitative analysis is beyond the scope of these experiments. However, some important observations can be drawn:

Bismuth (Bi) and B_2_O_3_ are the predominant products, consistent with eq 1 The absence of B_4_C in the stoichiometric composition and its persistence in the fuel-rich formulation, supported by the molar balancing of products, reinforces that the reaction equation is well-balanced.

The boron nitride (BN) formation was anticipated due to the presumed diffusion of N_2_ from the air into the reaction zone. When excluding BN as a possible combustion product, the amount of unreacted B_4_C increases and a small amount (0.15% in mass) of graphite (carbon) appears as a possible product. Further experimental verification of their formation will be discussed in later sections. The presence of hydrogen-containing species is attributed to the decomposition of PVA.

As expected, increasing fuel content led to a shift toward less oxidized species. The low CO_2_/CO ratios, even in the stoichiometric case, suggest that the software predicts that oxygen is largely consumed to form products like HBO_2_ rather than fully oxidizing carbon and hydrogen.

The percentage of solid residues collected exceeded the predicted value, likely due to the recondensation of vaporized Bi upon chamber cooling. Thermodynamic calculations suggest that Bi is the predominant gas-phase species at equilibrium. However, as the system cooled down, Bi vapor likely transitioned back to the solid phase, contributing to the higher residue recovery.

Following the analysis of pressure behavior and thermodynamic trends, FTIR spectroscopy was employed to characterize the collected gaseous products, offering a direct experimental insight into the reaction atmosphere and oxidation level of the system.

### 3.7 FTIR Analysis of Combustion Gases

Figure 10 compares the FTIR spectra of combustion chamber gases from stoichiometric (blue) and fuel-rich (red) tests, highlighting CO and CO_2_ as the main species, consistent with Horiuchi.^18^ Normalizing their absorbance to concentration using standard references yielded CO_2_/CO ratios of 0.67 (stoichiometric) and 0.34 (fuel-rich). However, a quantitative analysis is approximate and inherently imprecise, as potential contamination during gas collection and measurement could affect the results, given the challenge of maintaining a completely controlled internal atmosphere. A small fraction of hydrocarbon species (e.g., CH_4_, C_2_H_4_, and C_2_H_2_) was also identified in both samples.

**Figure 10:**
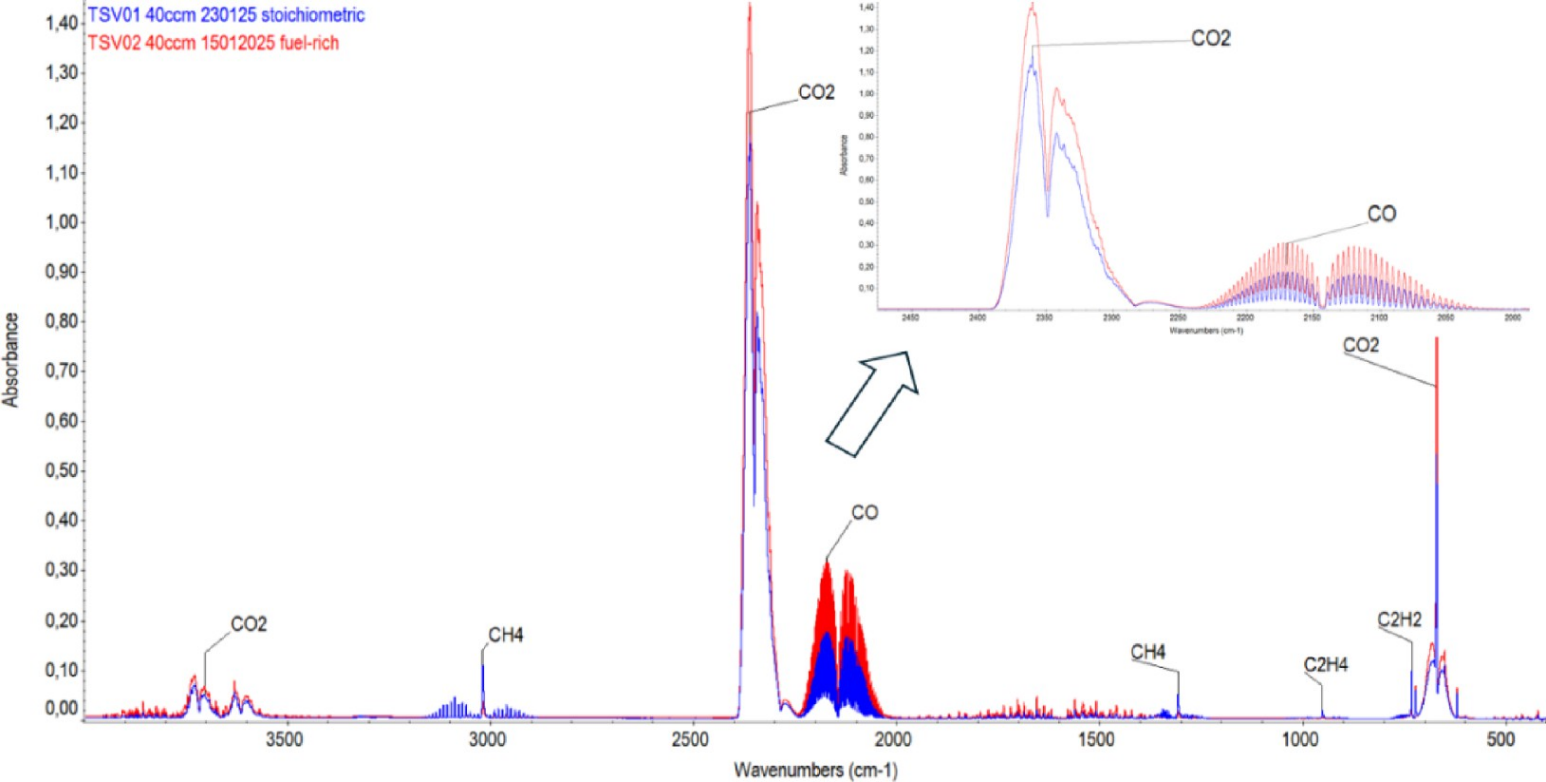
Comparative FTIR spectra of combustion gas compositions in a combustion chamber.

While experimental results indicate a higher CO_2_ presence than predicted, the overall trend aligns with expectations—higher fuel content shifts combustion products toward less oxidized species. Notably, the identified gaseous products are relatively simple and exhibit low toxicity compared to halogenated or metal-containing species commonly found in traditional pyrotechnic formulations, supporting the potential of this system as a more environmentally friendly alternative.

Since the reaction involves both condensed-phase reactants and products, characterizing the solid residues is essential to evaluate the extent of reaction and gain insight into the actual progression of the reaction pathway. For this, SEM imaging and EDS analysis were employed to investigate the morphology and elemental composition of the combustion residues.

### 3.8 SEM and EDS of Burning Residues

Figure 11 presents the SEM analysis of solid residues from the combustion of B4. Figure S5 exhibits images of the other samples. Figure 11a shows the top view of the burned delay element, revealing the distribution of residues across the surface, while Figure 11b provides a magnified view of a region rich in bright spherical structures. These spheres are likely bismuth that transitioned through a liquid or gaseous phase during the reaction and solidified upon cooling. This phenomenon of spherical formation due to phase transition is well-documented in the literature.^33^

**Figure 11:**
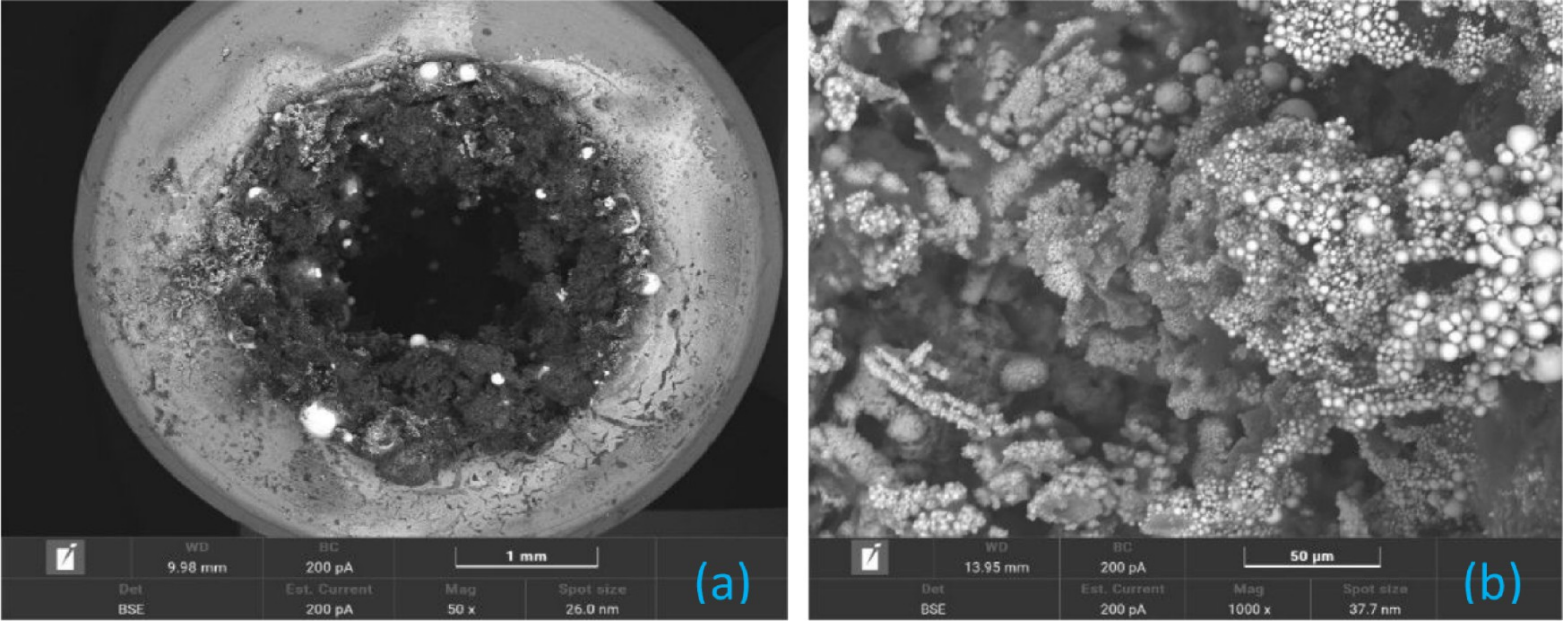
SEM analysis of B4 after burning: (a) top view (50 ×) of the burned delay element showing the distribution of solid residues across the surface; (b) magnified view (1 k×) of a region rich in bright spherical structures.

Figures 12 and 13 focus on specific regions of the burned composition at a 100 μm scale. These regions were selected for chemical composition analysis via EDS. Figure 12 highlights a larger spherical combustion product, while Figure 13 (and Figure S3, at higher magnifications) focuses on a cluster of smaller spheres, providing a comparative insight into their respective compositions.

**Figure 12:**
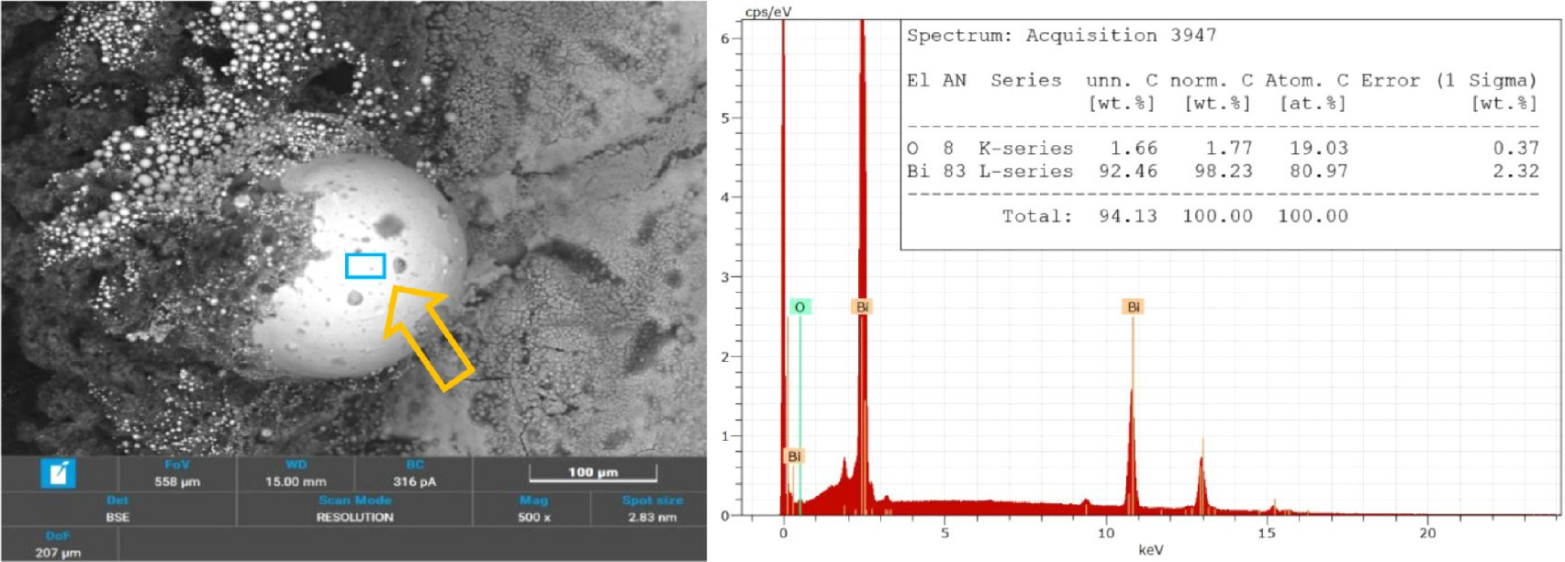
SEM image and EDS spectra of a larger sphere in B4 combustion residues.

**Figure 13:**
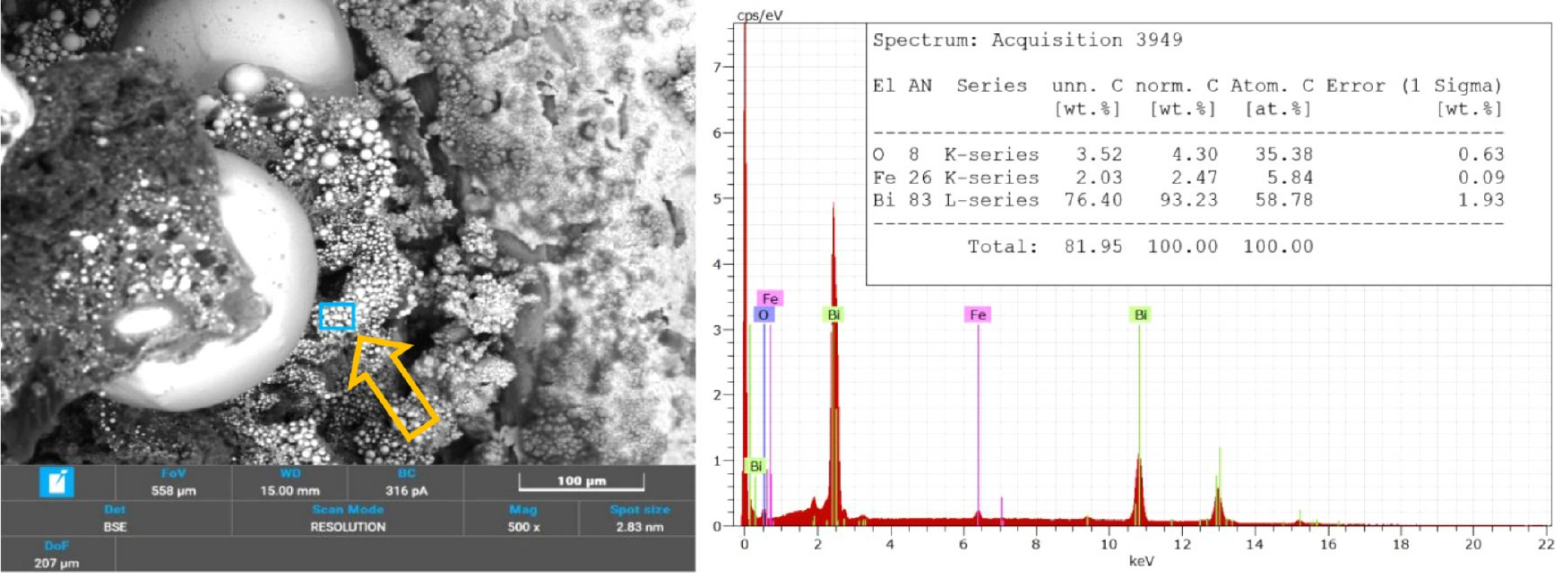
SEM image and EDS spectra of a cluster of small spheres in B4 combustion residues.

The EDS spectrum associated with Figure 12 confirms that the core of the larger spherical product primarily consists of Bi, the main combustion product predicted. This result aligns with the reaction outlined in eq 1 and is consistent with the expectations from BSE technique, where heavier materials appear as brighter regions. The small fraction of oxygen detected might correspond to both unreacted Bi_2_O_3_ or product B_2_O_3_, which are also plausible from the reaction. Boron, inherently challenging to detect due to its low atomic weight, may be present in quantities too small for accurate detection.^34^

The spectrum in Figure 13 reveals a slightly different composition. While Bi remains a major component, its relative concentration is lower compared to the larger sphere analyzed in Figure 12. Also, the presence of iron (Fe) is detected, likely originating from contamination caused by the combustion of the steel element. This contamination underscores the interaction between the combustion products and the surrounding materials.

To further investigate the presence of additional reaction products, additional EDS analyses were conducted on darker regions within the combustion residues, as shown in Figures 14 and 15. Since reliable quantitative detection is limited by EDS software constraints—it measures only surface layers and has reduced sensitivity to light elements (B, C)—boron and carbon were manually inspected. These regions exhibited significantly different elemental compositions compared to the bright spherical formations.

**Figure 14:**
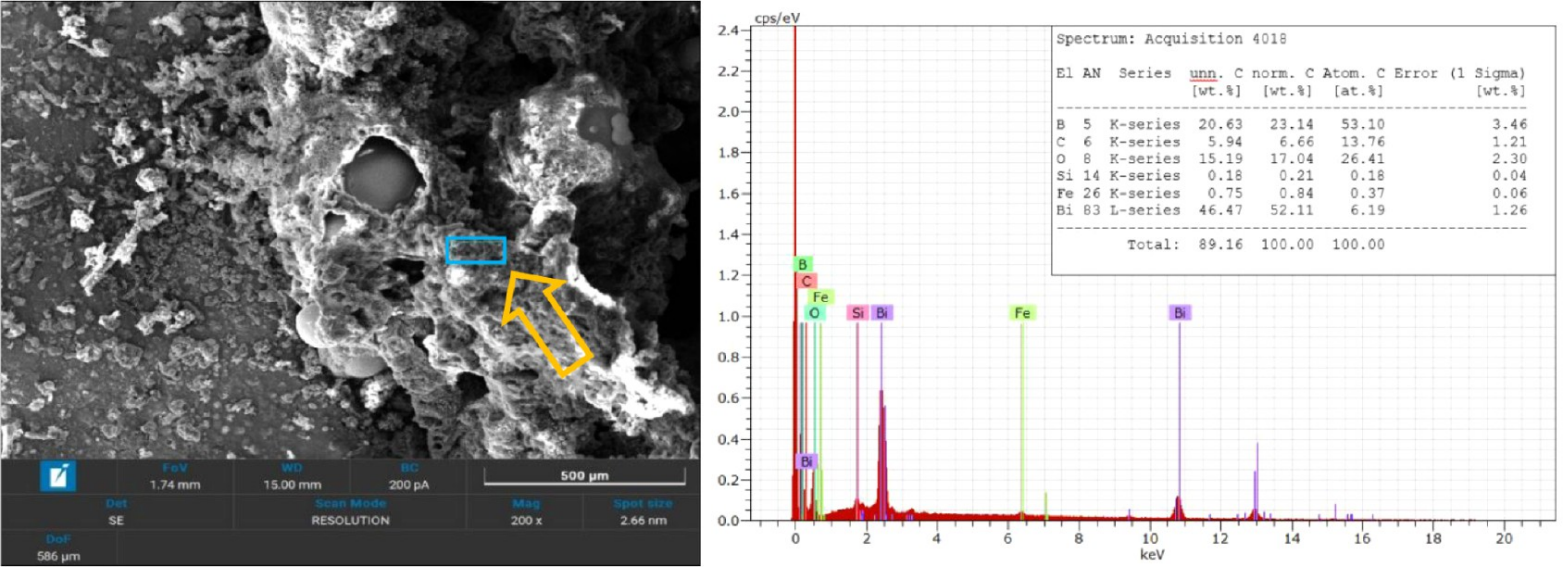
SEM image and EDS spectra of the first darker region in B4 combustion residues.

**Figure 15:**
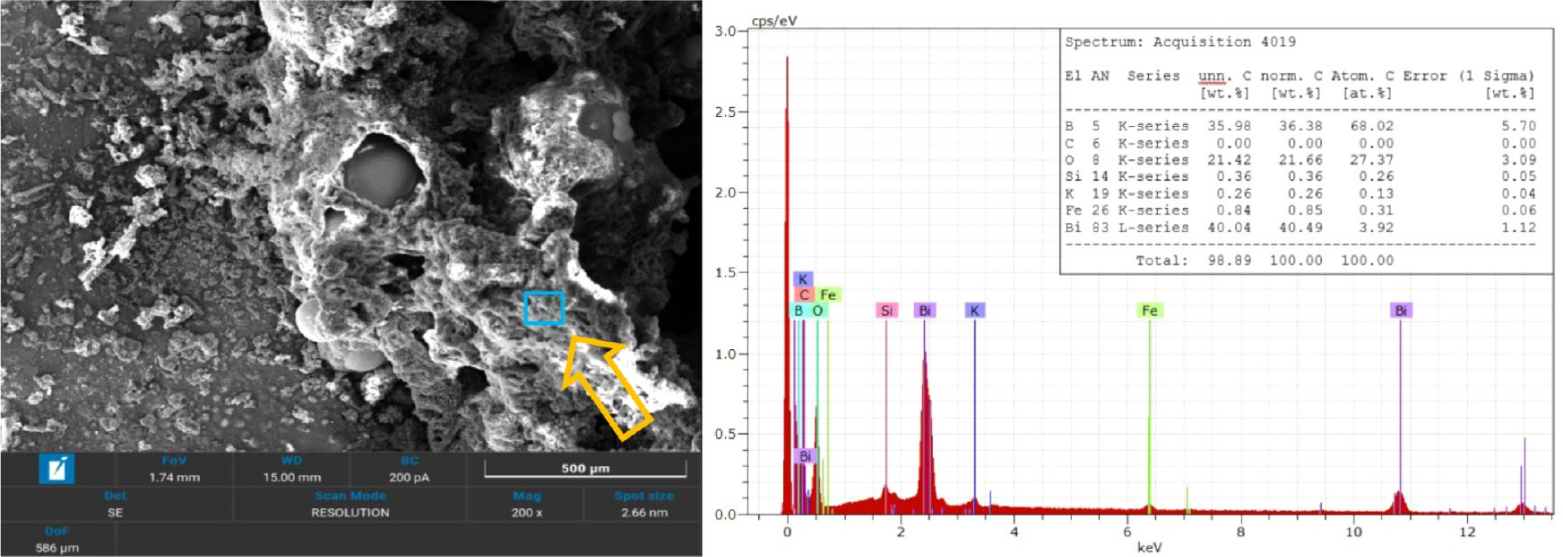
SEM image and EDS spectra of the second darker region in B4 combustion residues.

A key observation is the pronounced presence of boron and oxygen, which strongly suggests the formation of B_2_O_3_, a predicted combustion product both from eq 1 and thermodynamic calculations. The detection of carbon in some regions but not others suggests that traces of unreacted B_4_C may still be present. Another possibility is the presence of graphite. The variability in elemental distribution suggests heterogeneity in the residues. Additionally, unexpected potassium (K) and silicon (Si) appeared in the spectra, likely originating from contaminants.

Overall, the SEM/EDS findings confirm that the combustion process led to a complex and heterogeneous distribution of products, with expected elements appearing in different phases of the residue. The presence of bismuth in the brighter regions and boron with oxygen in the darker ones further reinforces product formation as outlined in eq 1.

Elemental mapping of the B4 combustion residues, shown in Figure 16, reveals a clear compositional contrast between brighter and darker regions, consistent with the previously discussed conclusions. The spherical bright areas are once again confirmed to correspond to Bi-rich domains. Boron and oxygen signals overlap in most regions, indicating the expected formation of boron oxide. Carbon is also detected in similar areas, suggesting either unreacted B_4_C or the presence of solid carbonaceous species such as graphite.

**Figure 16:**
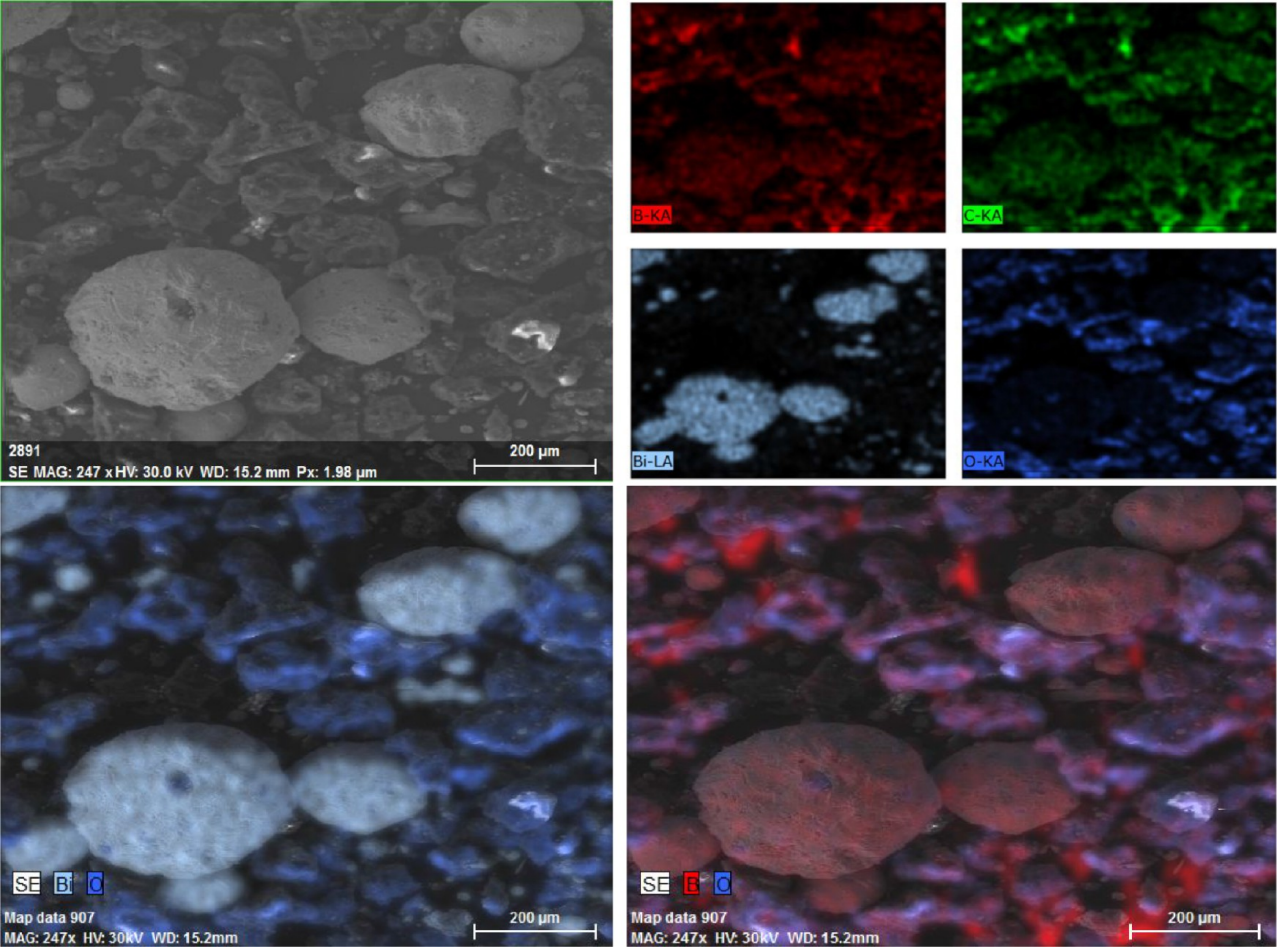
Elemental mapping of B4 combustion residues.

The superposition of boron, oxygen, and carbon signals is consistent with the formation of an amorphous boron oxide matrix partially incorporating unreacted B_4_C or carbonaceous residues. The liquid B_2_O_3_ formed during combustion likely entrapped small B_4_C particles or carbon species upon solidification, resulting in coincident elemental signals.

To complement the identification of combustion products, XRD and XPS analyses were performed on the solid residues. While XRD confirmed bulk-phase composition, XPS provided insights into the surface chemical states. Additional content is presented in Supporting Information.

### 3.9 XRD of Burning Residues

Figure 17 presents the XRD spectrum of the same residue of B4. The spectra of all the other residues are exhibited in Figure S9. The main peaks corresponding to the expected products, Bi and B_2_O_3_, exhibit a strong correlation with the reference data obtained from the XRD database (JCPDS cards). Notably, Tran reported the main characteristic peaks for B_2_O_3_ at 2θ = 14.5°, 27.9°, and 32.6°, further substantiating the identification of this phase.^35^

**Figure 17:**
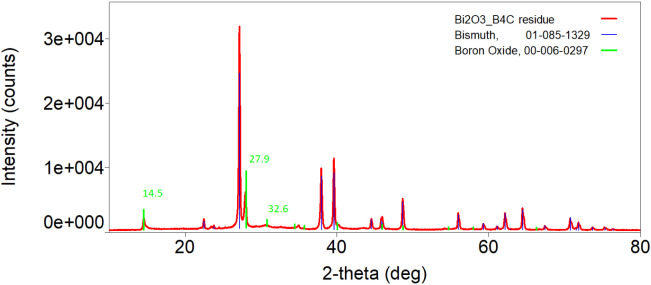
XRD spectra of the B4 combustion residues compared with the expected reaction products (Bi and B_2_O_3_, from JCPDS cards).

Comparison with the spectra of the initial reactants (Figures 18, S7 and S8) shows that Bi_2_O_3_ peaks are nearly absent, indicating its complete consumption in the reaction. Although thermodynamic predictions suggested the presence of excess B_4_C, XRD did not confirm this, likely due to structural changes, low concentration, or secondary reactions. The expected formation of BN and graphite was also not supported, as their characteristic peaks were not detected, suggesting limited nitrogen diffusion and no significant carbon segregation.

**Figure 18:**
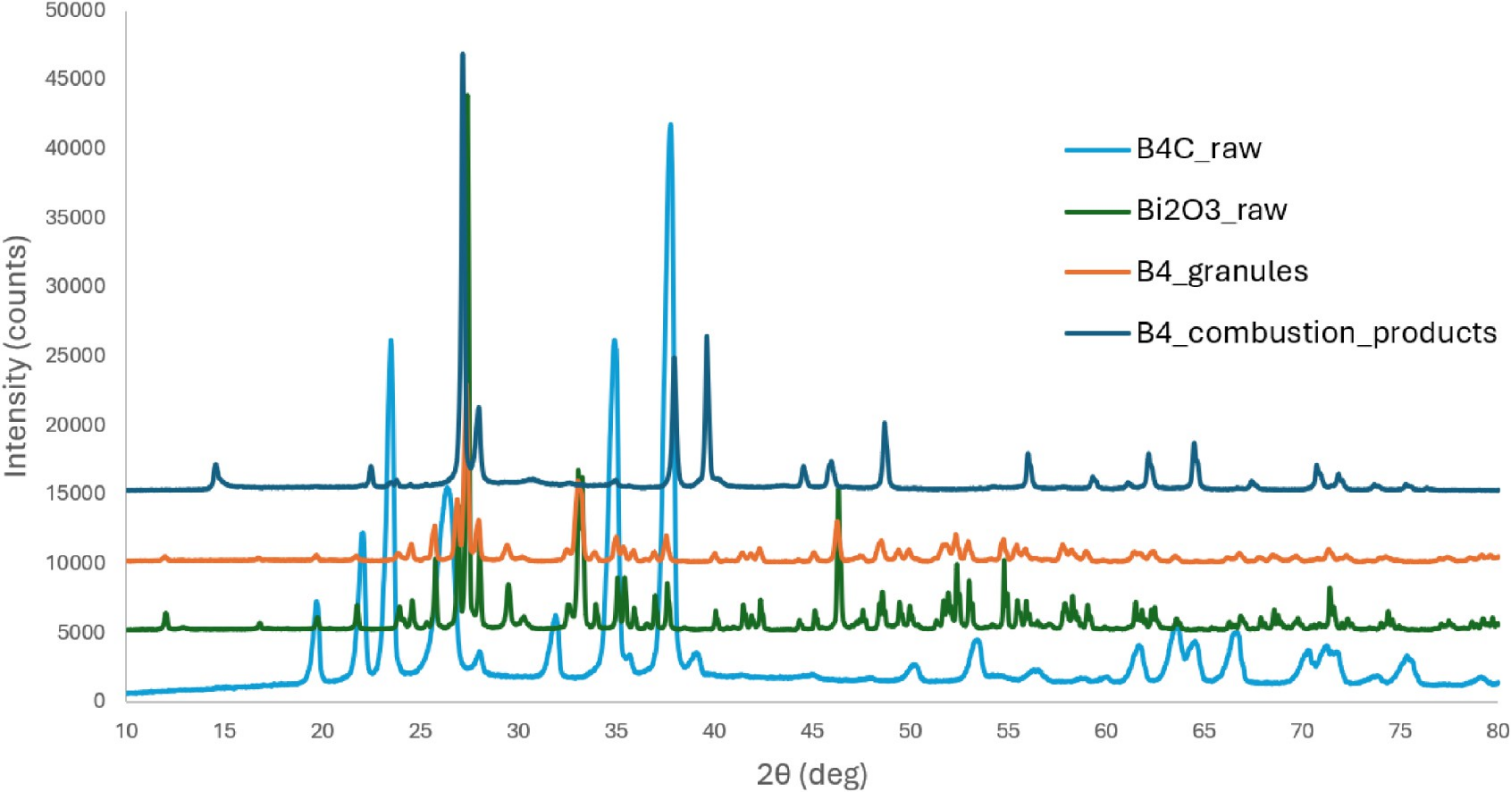
XRD spectra of the B4 combustion residues compared with the initial reactants (Bi_2_O_3_ and B_4_C powders and granules).

### 3.10 Surface Chemistry of Combustion Residues (XPS)

Survey spectrum scans identified the main photoelectron lines corresponding to carbon, oxygen, boron, and bismuth. High-resolution scans for the O 1s, C 1s, B 1s, and Bi 4f regions of B4 are presented in Figure 19, allowing detailed peak deconvolution and chemical assignment.

**Figure 19:**
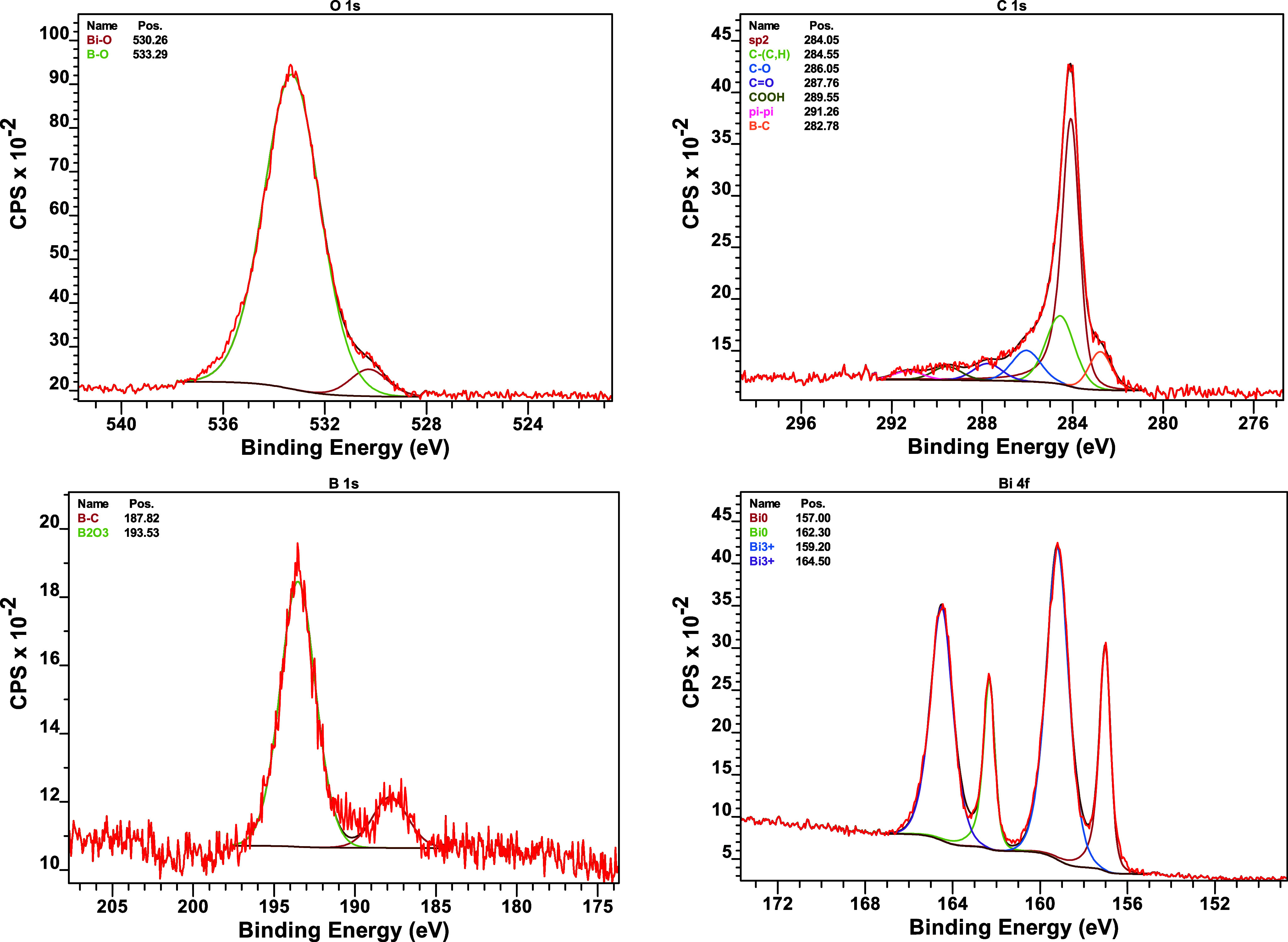
XPS high-resolution spectra of O 1s, C 1s, B 1s, and Bi 4f regions for B4.

The analysis confirmed B_2_O_3_ as a major surface product and indicated the presence of unreacted B_4_C in the B4 sample through B–C bonds. sp^2^ carbon was also detected in B4, suggesting possible graphite formation, as predicted in thermodynamic calculations. A2 showed more Bi^3+^ than B4, supporting that the stoichiometric composition had more residual Bi_2_O_3_, while the fuel-rich B4 sample promoted greater reduction to Bi^0^. The presence of Bi^3+^ on the surface observed may result from postcombustion reoxidation of Bi^0^. These results reinforce the proposed reaction pathway and the role of fuel excess in enhancing reaction completeness. In this analysis, the B 1s spectrum further confirms the absence of BN by the lack of B–N peaks, consistent with the EDS results, which also revealed no detectable nitrogen signal.

All experimental findings contribute to the fundamental understanding of boron carbide and bismuth oxide reactions under both basic and applied conditions, particularly in delay compositions. These results provide new insights into the reaction pathway and the underlying combustion mechanism.

## 4 Conclusions

Stoichiometric formulations of B_4_C/Bi_2_O_3_, which ignited when not pressed, failed to burn after being lightly compacted; a fuel-rich composition is necessary to ensure combustion. The fuel-rich compositions ignited readily and maintained combustion without quenching. Water facilitated the granulation process and improved the final composition. With increasing loading pressure, burning rates decrease due to reduced convective heat transfer, leading the combustion process to transition toward a conductive regime. Particle size also impacted combustion, particularly with larger Bi_2_O_3_ particles, which influenced the combustion profile up to a certain threshold. Additionally, the granule size of the final composition was found to alter the burning behavior, with larger granules promoting faster combustion at lower pressures.

Thermodynamic calculations provided an initial insight into the reaction process. The findings show that the reaction progresses through distinct stages: starting in a solid–solid preignition phase, transitioning to a solid–liquid phase as components melt, and culminating in a gas phase marked by significant product volatilization, illustrating a complete combustion evolution.

FTIR analysis confirmed that the main gaseous products are CO and CO_2_, indicating reduced environmental toxicity. Elemental and phase analyses confirmed that the reaction followed the expected pathway, with Bi and B_2_O_3_ as the predominant products. No Bi_2_O_3_ was detected by XRD, indicating that the oxidizer was fully consumed in the bulk. Morphological analysis of the products suggests that, upon cooling, bismuth solidifies into small metallic spheres, while surface analysis indicates that B_2_O_3_ remains predominantly in the outer layer. Altogether, the results are consistent and complementary, reinforcing the reliability and scientific rigor of the study.

All these observations accentuate the necessity of conducting experiments under application-specific conditions. These findings not only enhance our understanding of the combustion dynamics of low toxicity B_4_C/Bi_2_O_3_ mixtures but also underscore their potential for practical applications requiring precise delay times and environmental sustainability. The successful use of water as a green solvent further highlights the viability of developing sustainable pyrotechnic compositions. This research advances the field by providing valuable insights into optimizing performance while minimizing environmental impact through careful material selection and process control.

## Supplementary Material



## Notes

Additional SEM micrographs of unpressed granules and combustion residue samples; additional elemental mapping and EDS spectrum of unpressed granule samples; additional XRD patterns of combustion residues; additional XPS patterns of combustion residues (PDF)
