# Supplementary material for: Sustainable Pyrotechnics: Combustion Behavior of B4C/Bi2O3 for Delay Compositions
Source: ACS Omega. 2026 Jan 12;11(3):4668–83. doi: 10.1021/acsomega.5c10964 (PMC12854626; doi:10.1021/acsomega.5c10964)
Supplement: Supplementary file 1 [file ao5c10964_si_001.pdf]

Sustainable Pyrotechnics: Combustion Behavior of B4C/Bi2O3 for Delay Compositions

Danillo F. V. Cantini,[1] Vojtěch Pelikán*[1], Eva Schmidová[2] and Jiří Pachman[1]

[1] Institute of Energetic Materials, Faculty of Chemical Technology, University of Pardubice, Studentská 95, 532 10 Pardubice, Czech Republic

[2] Educational and Research Centre in Transport, Faculty of Transport Engineering, University of Pardubice, Studentská 95, 532 10, Pardubice, Czech Republic

* vojtech.pelikan@upce.cz

SEM. Figure S1 illustrates higher magnifications of B4 granules.

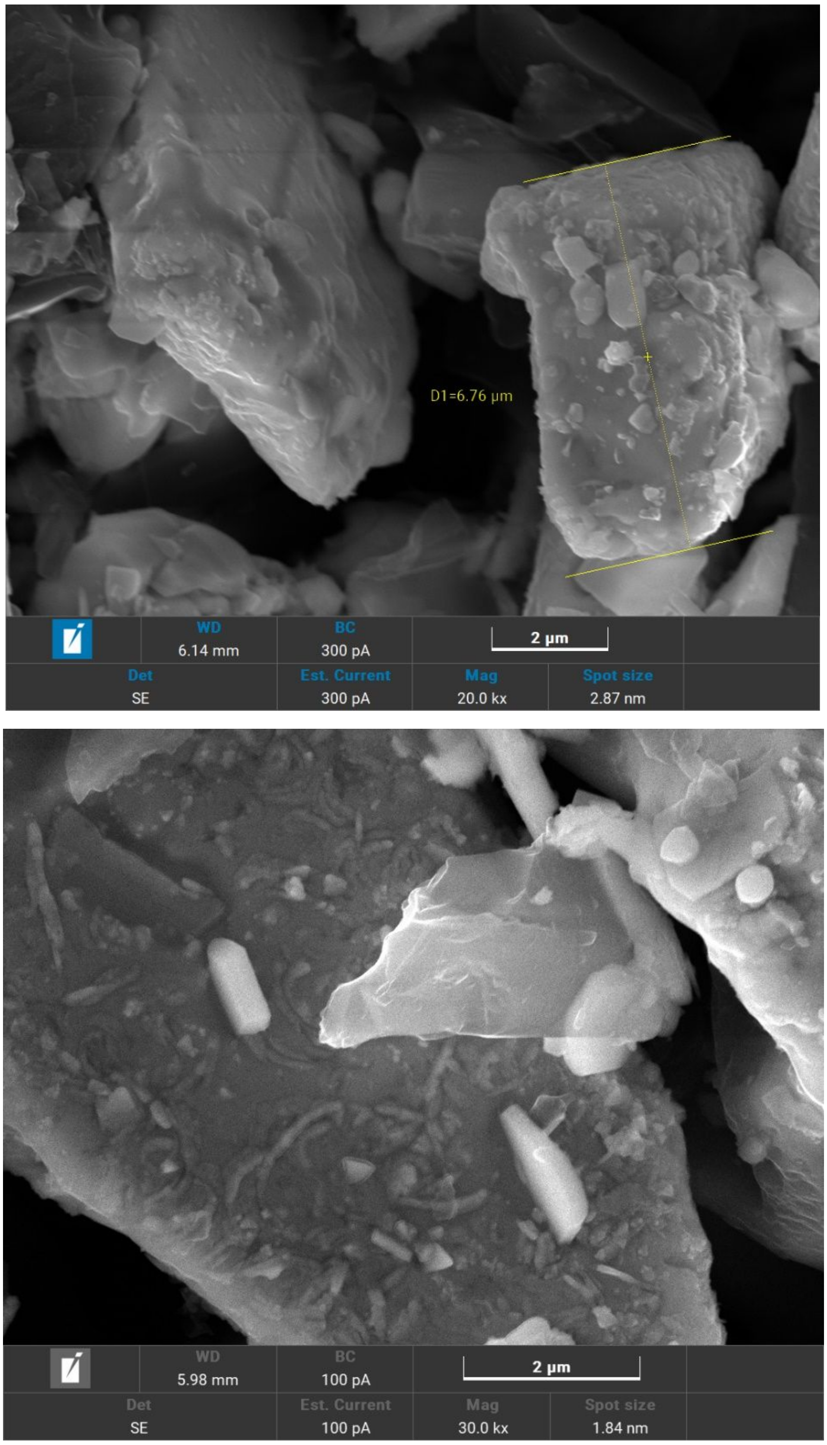

Figure S1: Higher magnifications of B4 granules before pressing.

Figure S2 depicts the elemental mapping (and EDS) of B4 granules. It can be seen that the darker regions correspond to boron, while the brighter ones, to the bismuth oxide.

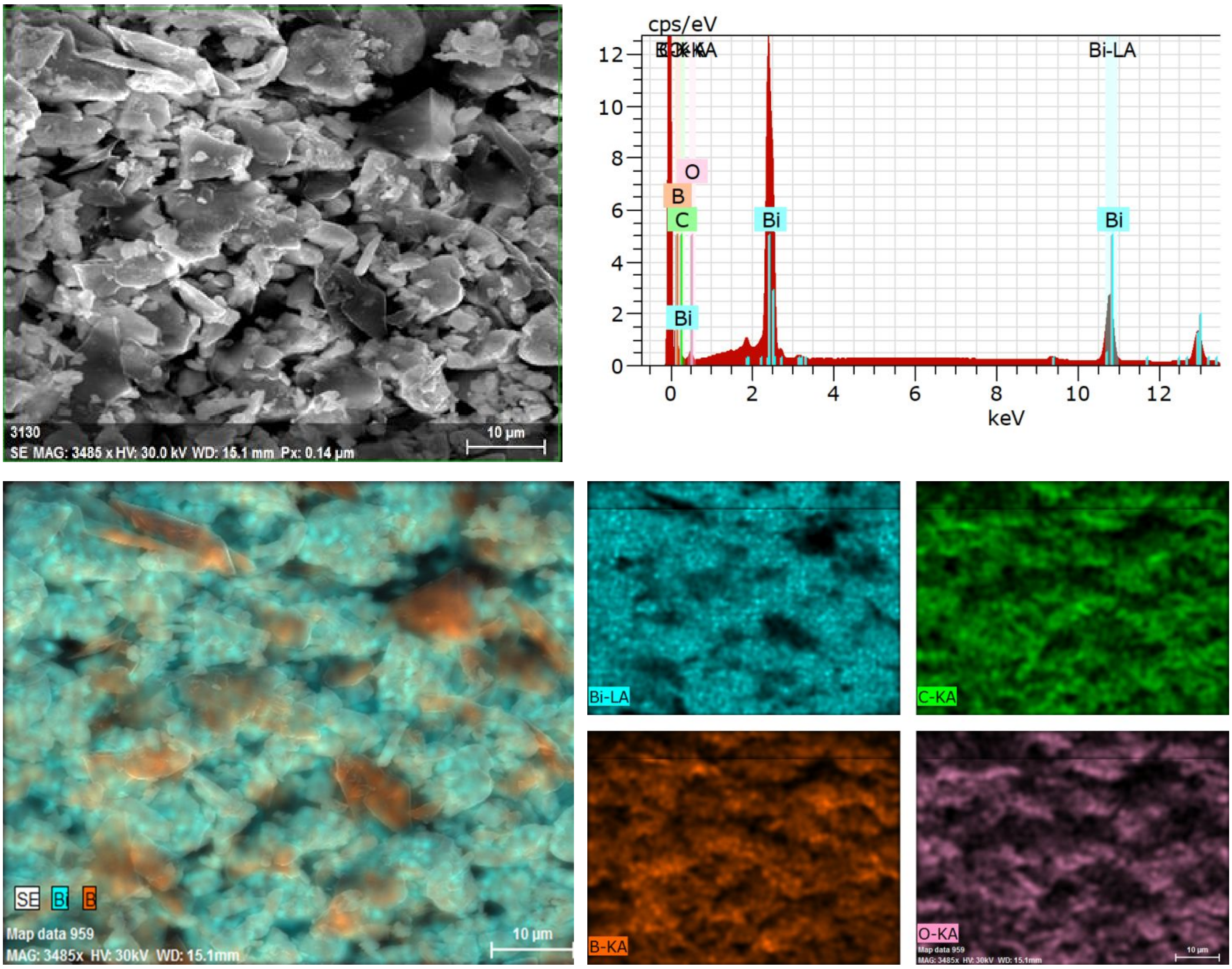

Figure S2: Elemental mapping and EDS spectrum of B4 granules before pressing.

Figure S3 illustrates higher magnifications of B4 combustion residues next to a cluster of small spheres (bismuth).

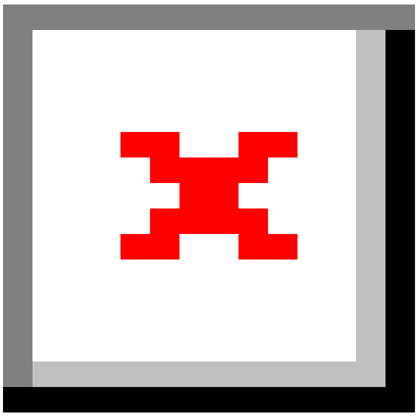

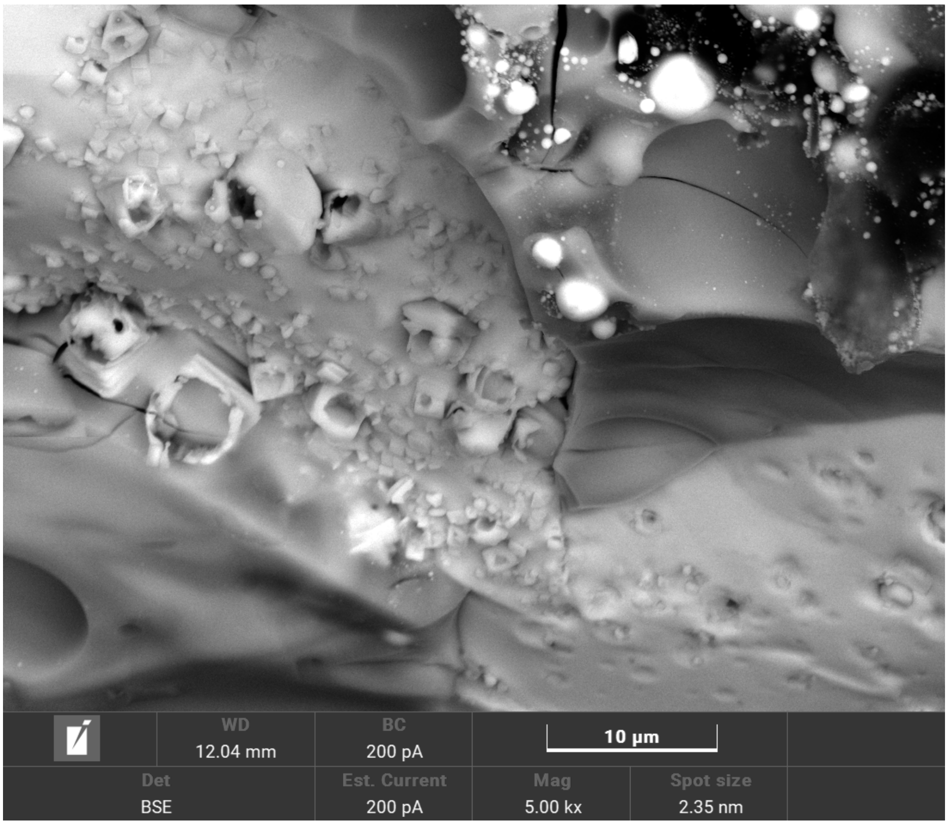

Figure S3: Higher magnification of B4 combustion residues next to a cluster of bismuth spheres.

Figure S4 illustrates higher magnifications of B4 combustion residues next to the flat and darker areas.

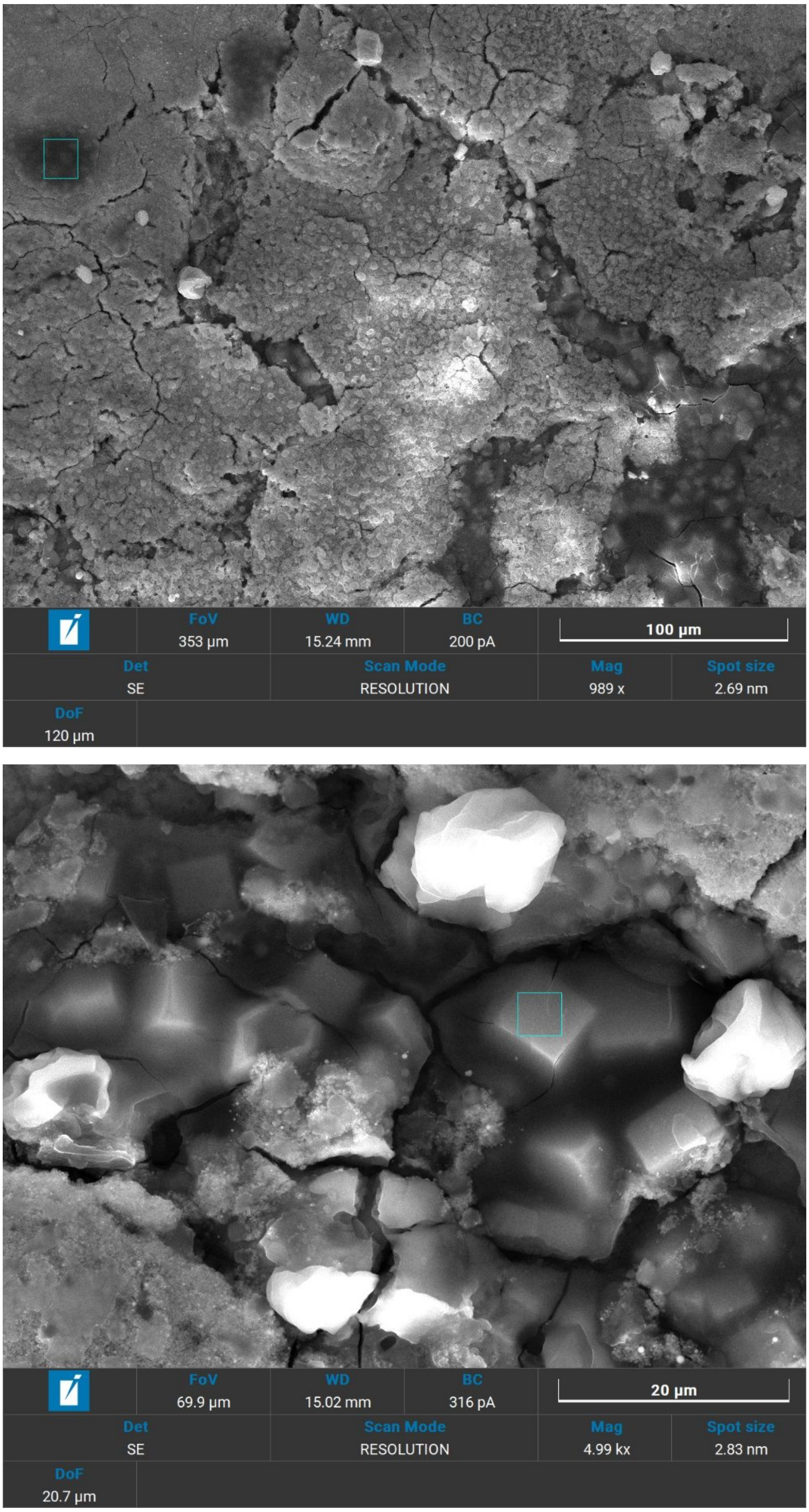

Figure S4: Higher magnification of B4 combustion residues next to the flat and darker areas.

Figure S5 displays the combination of batches B3 to B6 in 1000 × magnification before (top line) and after (bottom line) the burning.

B3 *B4* *B5* *B6*

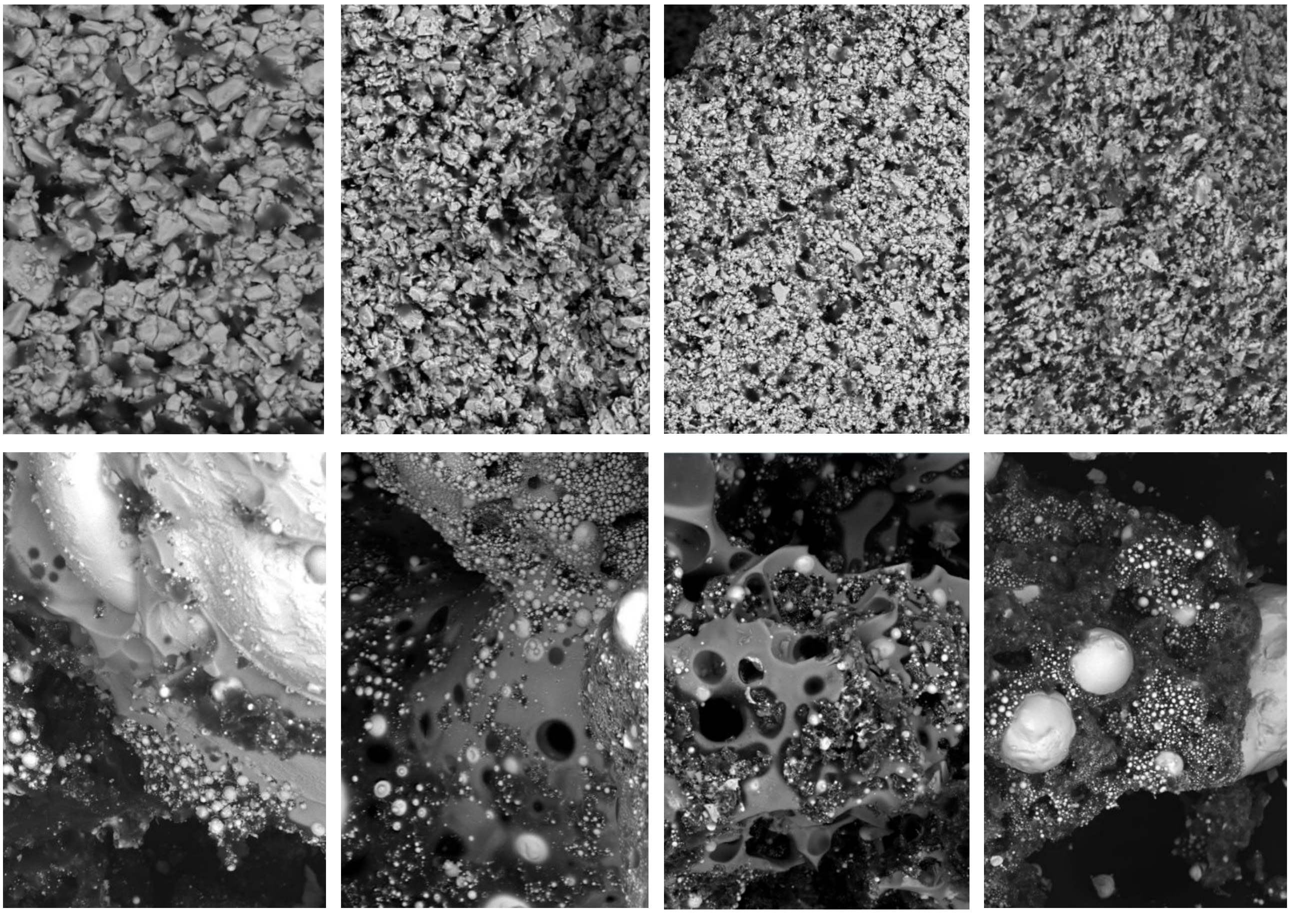

Figure S5: Comparison of combustion residues from batches B3, B4, B5 and B6 in magnification of 1000 ×.

XRD of Burning Residues. Figure S6, Figure S7, and Figure S8, present the XRD spectra of the post-reaction residue of B4, providing complementary information for the material characterization.

In Figure S6, the main peaks corresponding to the expected products, Bi and B_2O_3, exhibit a strong correlation with the reference data obtained from the XRD database. Notably, Tran reported the main characteristic peaks for B_2O_3 at 2θ = 14.5, 27.9, and 32.6°, further substantiating the identification of this phase.[1]

Additionally, in Figure S7 and Figure S8, the spectrum of the product was compared with the spectra of the initial reactants, including the granules formed after granulation. The characteristic peaks of Bi_2O_3, which distinguish it from pure Bi, are almost absent. This supports the conclusion that Bi_2O_3 was entirely consumed, as expected in a fuel-rich reaction. Regarding B_4C, thermodynamic simulations predicted an excess of this material in the products. However, the XRD analysis does not provide direct evidence to confirm its presence. It is possible that structural changes during high temperature combustion altered its crystallinity, making its peaks shifted or undetectable by XRD. The fact that it is present in very small amounts also contributes to its non-detection. Alternatively, this absence could indicate that B_4C was consumed in secondary reactions, forming different compounds.

BN was predicted in thermodynamic calculations, but its most prominent peak (2θ = 26.3°)[2] was not detected in the peak list, suggesting it was not formed. The software assumes complete mixing of air with the sample, enabling rapid reactions. However, the absence of BN indicates that N_2 likely did not diffuse sufficiently to react with boron species. Similarly, graphite formation is not supported by this technique, as its strongest characteristic peak (2θ = 26.5°)[3] is also absent in the peak list.

The confirmation that Bi_2O_3 was fully consumed, with Bi and B_2O_3 as the primary products, aligns with the expected reaction pathway, reinforcing the consistency and reliability of the combustion process. While a precise quantitative analysis remains challenging, the results qualitatively support that the reaction proceeded as outlined in Equation 1.

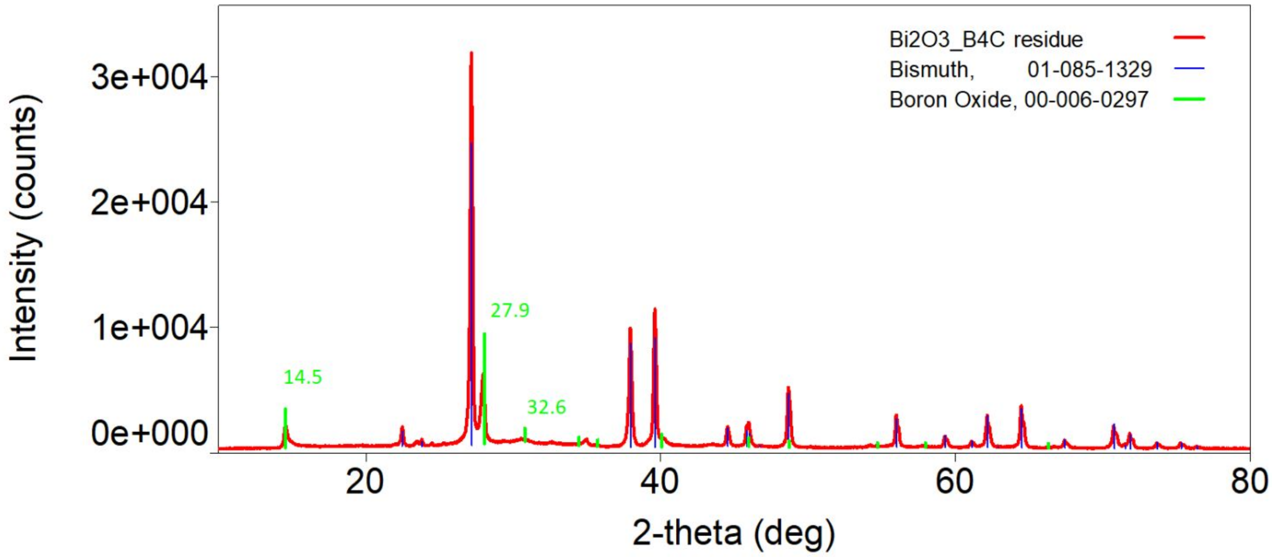

Figure S6: XRD spectra of the post-reaction residue showing the main products (Bi and B_2O_3).

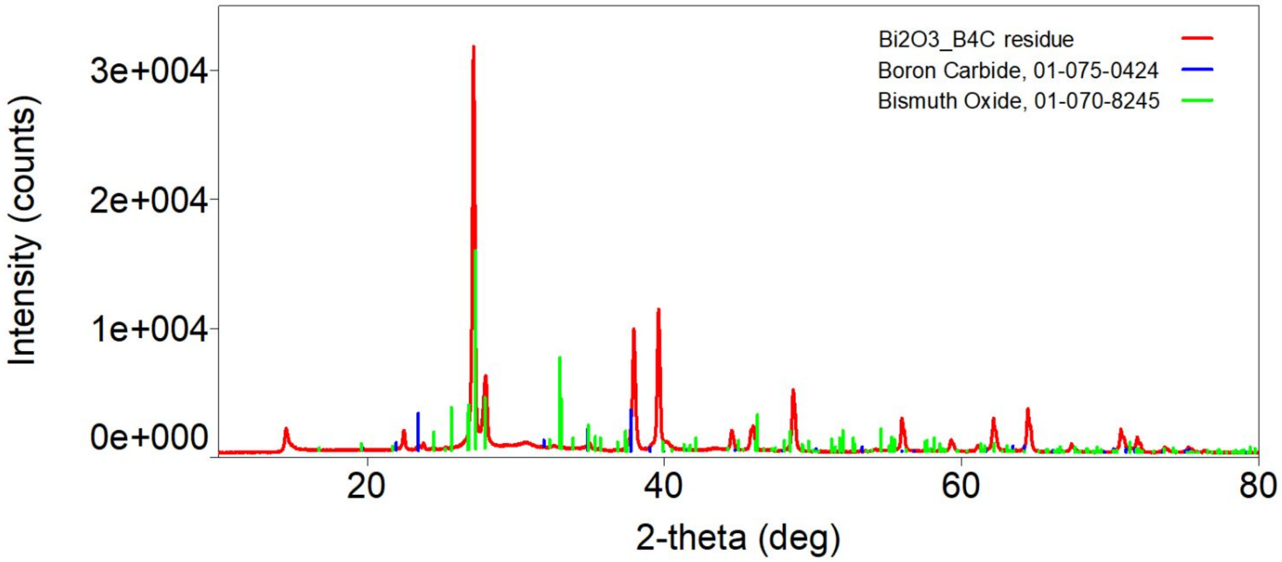

Figure S7: XRD spectra of the post-reaction residue showing the comparison with the initial reactants (Bi2O3 and B4C) from JCPDS cards.

Figure S9 displays the XRD patterns of the combustion residues from other batches. The results are very similar, although the peaks corresponding to B_2O_3 are barely visible. This suggests that the combustion process promoted the formation of an even less crystalline boron oxide phase, or alternatively, the generation of boron suboxides, which exhibit a characteristic reflection near $2\theta = 33^\circ$.

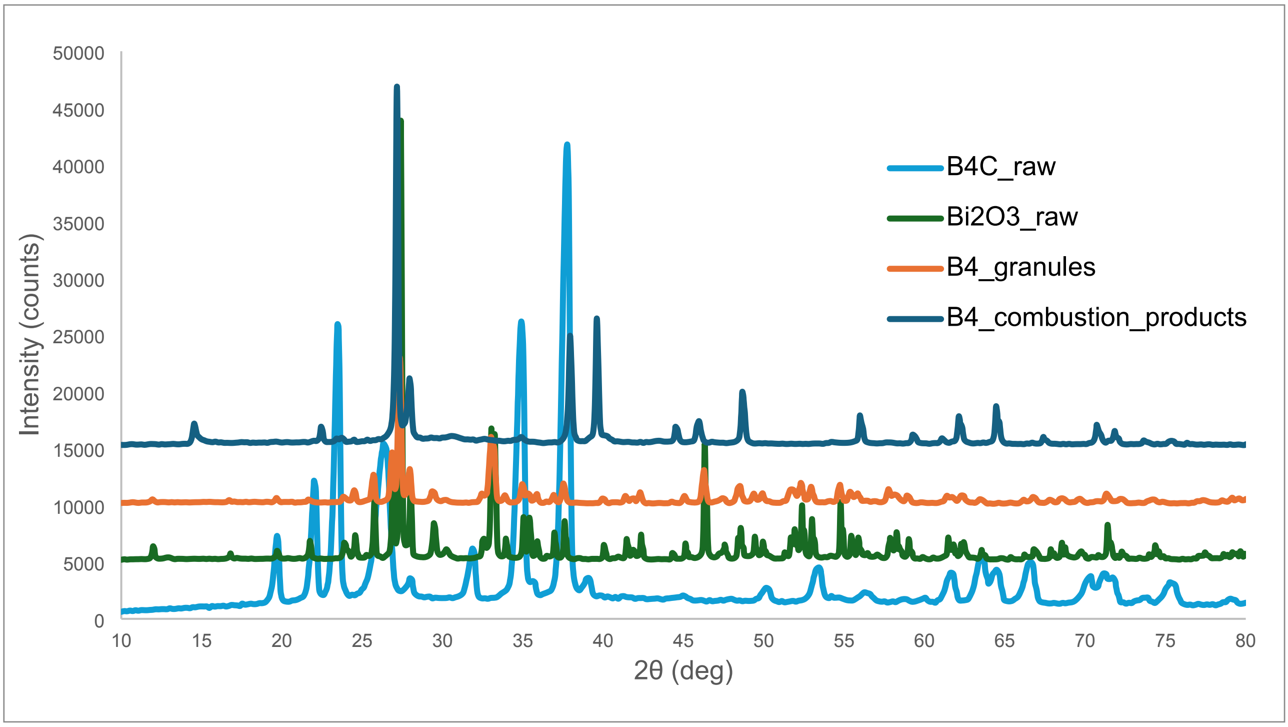

Figure S8: XRD spectra of the post-reaction residue of B4 showing the comparison with the initial reactants (Bi_2O_3 and B_4C) and the granules before combustion, from experimental measurement.

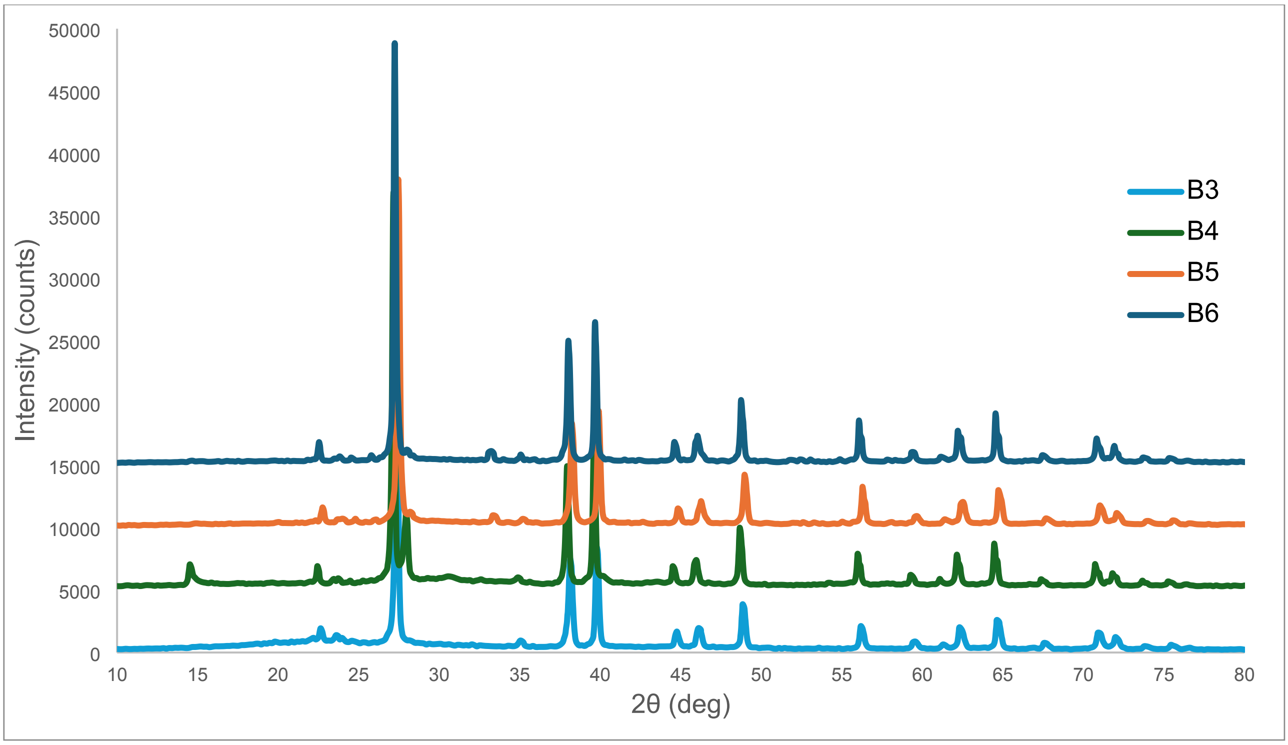

Figure S9: XRD comparison of batches combustion products.

To complement the SEM/EDS and XRD analyses of the solid residues, XPS was performed to investigate the surface chemical states and binding environments of key elements. Two representative samples were selected: A2 (stoichiometric), and B4 (fuel-rich).

Surface Chemistry of Combustion Residues (XPS).

The survey spectrum of B4 is presented in Figure S10, showing the main photoelectron lines corresponding to carbon, oxygen, boron, and bismuth. High-resolution scans were also performed for the O 1s, C 1s, B 1s, and Bi 4f regions (Figure S11), allowing detailed peak deconvolution and chemical assignment.

Table S1 presents the atomic concentrations and the corresponding binding energies (eV) for the main chemical species identified in the spectra of A2 and B4.

The C 1s region of B4 showed distinct contributions from sp^2 carbon (15.56%), not detected in A2, likely suggesting the formation of graphite-like structures during combustion, possibly due to thermal decomposition of B_4C or surface carbonization. B–C is present both in C 1s and B 1s spectra, corresponding to 4.17% atomic concentration, supporting the presence of unreacted boron carbide in the residue. This result is consistent with incomplete conversion under certain fuel-rich conditions, where excess B_4C may remain unoxidized, aligning with thermodynamic calculations predictions.

B 1s spectrum also indicates strong B_2O_3 peaks in both A2 and B4, further confirming the formation of boron oxide as a dominant product. In B4, B–O accounted for 40.10% and B_2O_3 for 21.75% of the atomic surface composition, in agreement with the expected reaction products and consistent with XRD identification of B_2O_3. Although bismuth may volatilize and recondense, it likely deposits in localized or subsurface regions, limiting its detection by the shallow XPS probe. In contrast, B_2O_3 forms mainly continuous amorphous films that remain on the surface, leading to higher apparent concentrations.[4] In this analysis, the B 1s spectrum further confirms the absence of BN by the lack of B–N peaks, consistent with the EDS results, which also revealed no detectable nitrogen signal.

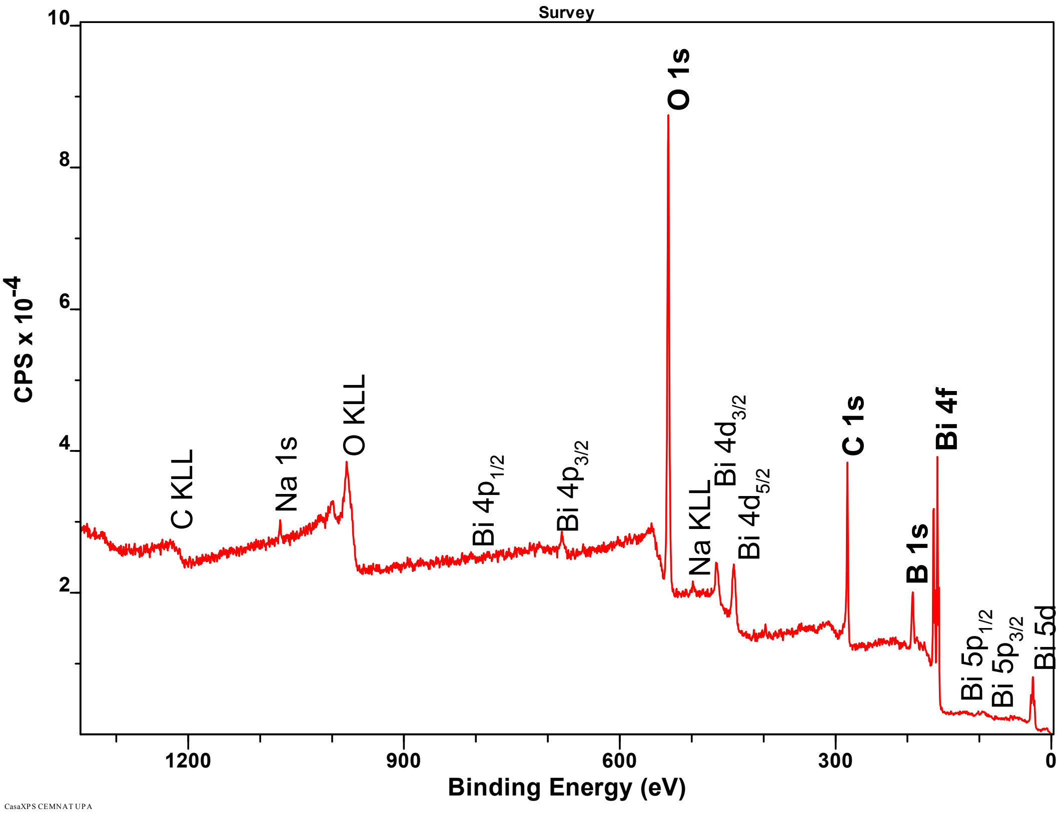

Figure S10: XPS survey spectrum for B4.

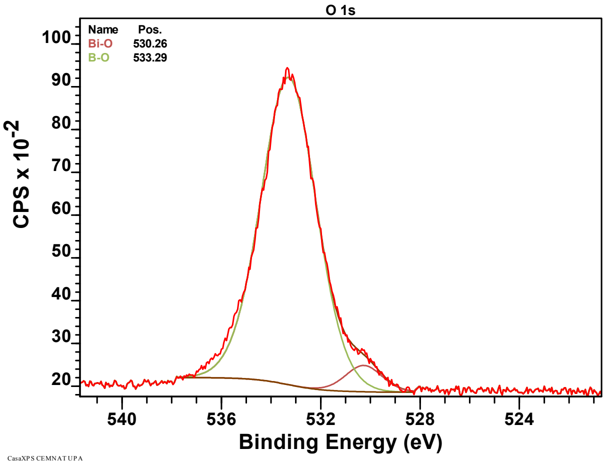

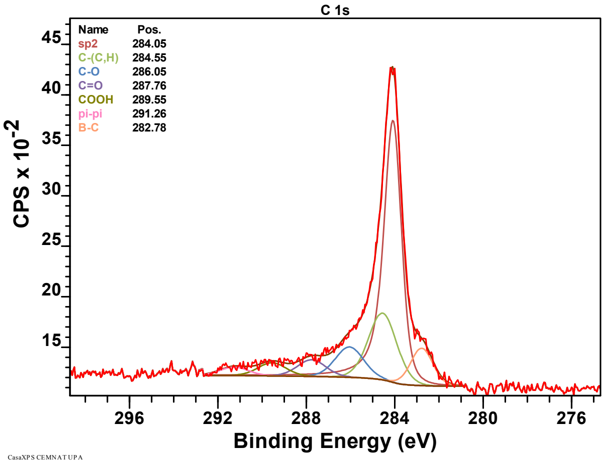

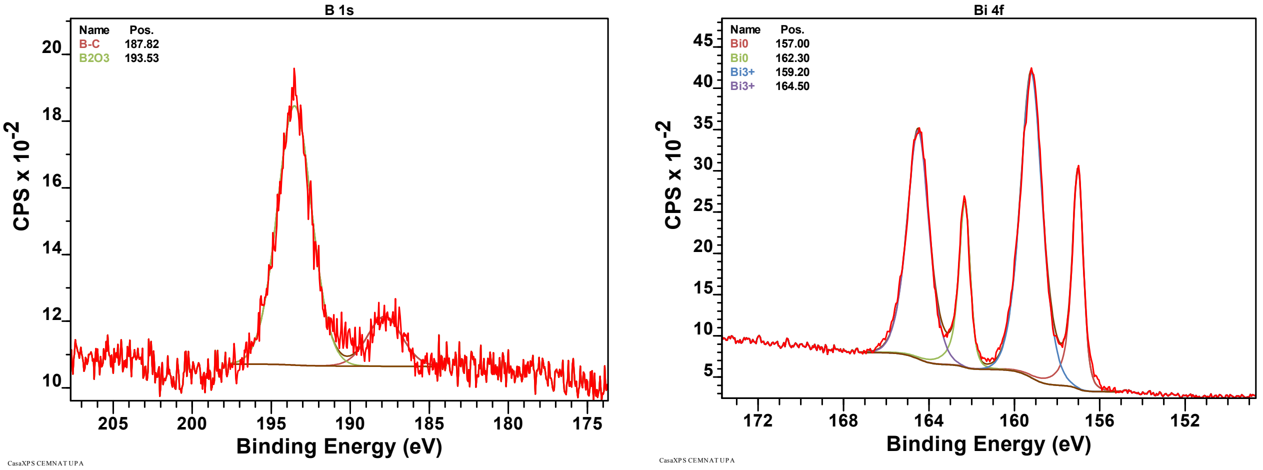

Figure S11: XPS high resolution spectra of O 1s, C 1s, B 1s, and Bi 4f regions for B4.

A comparison between the Bi 4f spectra of A2 and B4 revealed significant differences in the relative contributions of Bi^0 and Bi^{3+}. The stoichiometric sample A2 showed a higher proportion of Bi^{3+} (5.57%) compared to B4 (1.70%), while Bi^0 remained similar in both. This suggests that in the stoichiometric condition, more Bi_2O_3 remained at the surface, whereas in the fuel-rich composition, Bi_2O_3 was more completely reduced to Bi^0. However, it must be noted that XPS is a surface-sensitive technique, and Bi^0 exposed to air may undergo partial reoxidation, potentially affecting the measured ratio and explaining the remaining Bi_2O_3. This observation supports the notion that excess fuel promotes more extensive reduction of the oxidizer, resulting in a higher degree of reaction completeness.

Table S1: Surface chemical composition and binding energy positions in A2 and B4 residues by XPS.

	Sample	sp^2	C-(C,H)	C–O	C=O	COOH	B–C	Bi–O	B–O	Bi^0	Bi^{3+}	B–C	B_2O_3	Na^+
**A2**	**Atom. conc. (%)**	**0**	**9.35**	**2.20**	**1.50**	**0.76**	**0**	**2.54**	**51.79**	**0.72**	**5.57**	**0**	**25.56**	**0**
	Position (eV)	284.1	284.6	286.1	287.8	289.6	282.8	530.3	533.3	157	162.3	159.2	164.5	187.8
**B4**	**Atom. conc. (%)**	**15.56**	**5.47**	**2.49**	**1.37**	**1.08**	**2.40**	**2.28**	**40.1**	**0.71**	**1.70**	**4.17**	**21.75**	**0.92**
	Position (eV)	–	284.5	286	287.6	289.3	–	530.4	532.9	157	162.3	159.7	165	–

References.

(1) Tran, B. H.; Tieu, K.; Wan, S.; Zhu, H.; Cui, S.; Wang, L. Understanding the Tribological Impacts of Alkali Element on Lubrication of Binary Borate Melt. *RSC Adv.* **2018**, *8* (51), 28847–28860. https://doi.org/10.1039/C8RA04658A.

(2) Türkez, H.; Arslan, M. E.; Sönmez, E.; Açikyildiz, M.; Tatar, A.; Geyikoğlu, F. Synthesis, Characterization and Cytotoxicity of Boron Nitride Nanoparticles: Emphasis on Toxicogenomics. *Cytotechnology* **2019**, *71* (1), 351–361. https://doi.org/10.1007/s10616-019-00292-8.

(3) Destyorini, F.; Irmawati, Y.; Hardiansyah, A.; Widodo, H.; Yahya, I. N. D.; Indayaningsih, N.; Yudianti, R.; Hsu, Y.-I.; Uyama, H. Formation of Nanostructured Graphitic Carbon from Coconut Waste via Low-Temperature Catalytic Graphitisation. *Eng. Sci. Technol. Int. J.* **2021**, *24* (2), 514–523. https://doi.org/10.1016/j.jestch.2020.06.011.

(4) University of Reading; Wright, A. C. Review: The Structural Chemistry of B2O3. *Phys. Chem. Glas. Eur. J. Glass Sci. Technol. Part B* **2018**, *59* (2), 65–87. https://doi.org/10.13036/17533562.59.2.034.
